# Molecular architecture of the Gα_i_-bound TRPC5 ion channel

**DOI:** 10.1038/s41467-023-38281-3

**Published:** 2023-05-03

**Authors:** Jongdae Won, Jinsung Kim, Hyeongseop Jeong, Jinhyeong Kim, Shasha Feng, Byeongseok Jeong, Misun Kwak, Juyeon Ko, Wonpil Im, Insuk So, Hyung Ho Lee

**Affiliations:** 1 Department of Chemistry, College of Natural Sciences, Seoul National University, Seoul, 08826 Republic of Korea; 2 Department of Physiology, College of Medicine, Seoul National University, Seoul, 03080 Republic of Korea; 3 Center for Research Equipment, Korea Basic Science Institute, Chungcheongbuk-do, 28119 Republic of Korea; 4 Department of Biological Sciences and Chemistry, Lehigh University, Bethlehem, PA 18015 USA; 5 Present Address: Department of Physiology, University of California, San Francisco, San Francisco, CA 94158 USA

**Keywords:** Cryoelectron microscopy, Transient receptor potential channels

## Abstract

G-protein coupled receptors (GPCRs) and ion channels serve as key molecular switches through which extracellular stimuli are transformed into intracellular effects, and it has long been postulated that ion channels are direct effector molecules of the alpha subunit of G-proteins (Gα). However, no complete structural evidence supporting the direct interaction between Gα and ion channels is available. Here, we present the cryo-electron microscopy structures of the human transient receptor potential canonical 5 (TRPC5)-Gα_i3_ complexes with a 4:4 stoichiometry in lipid nanodiscs. Remarkably, Gα_i3_ binds to the ankyrin repeat edge of TRPC5 ~ 50 Å away from the cell membrane. Electrophysiological analysis shows that Gα_i3_ increases the sensitivity of TRPC5 to phosphatidylinositol 4,5-bisphosphate (PIP_2_), thereby rendering TRPC5 more easily opened in the cell membrane, where the concentration of PIP_2_ is physiologically regulated. Our results demonstrate that ion channels are one of the direct effector molecules of Gα proteins triggered by GPCR activation–providing a structural framework for unraveling the crosstalk between two major classes of transmembrane proteins: GPCRs and ion channels.

## Abstract (web summary)

Ion channels could be directly regulated by the alpha subunit of heterotrimeric G-proteins. Here, authors present the cryo-EM structure of an ion channel-Gα complex, TRPC5-Gα_i3_, and present functional insights into their direct interaction.

## Introduction

G-protein coupled receptors (GPCRs) represent one of the most fundamental and clinically relevant molecular switches converting extracellular stimuli into intracellular signals^1^. The first event that occurs following the activation of GPCRs by an extracellular agonist is the activation of heterotrimeric G-proteins; the activation of GPCRs promotes an alpha subunit (Gα) of a heterotrimeric G-protein to exchange a nucleotide from GDP to GTP inside its pocket, thereby triggering the dissociation of a heterotrimeric G-protein (Gαβγ) into Gα and Gβγ. Once activated, Gα proteins amplify the initial signal from the switch by activating effector molecules—mostly membrane-delimited enzymes—such as adenylyl cyclase or phospholipase C (PLC).

Owing to the comparable abundance of ion channels in the plasma membrane^2^ (1 channel/μm^2^, 100 GPCR/μm^2^, 1000 Gα/μm^2^), a number of lines of evidence suggest that not only membrane-delimited enzymes but also ion channels could also be considered direct effector molecules of Gα and Gβγ proteins^3,4^. In a sense, a possibility is that ion channels and GPCRs may be in the vicinity of one another and constitute a signaling cluster within a local area of the plasma membrane^2,5^. In fact, direct communication between ion channels and Gβγ was shown by crystal structure of the mammalian G-protein-activated inwardly rectifying potassium 2 (GIRK2) channel in complex with Gβγ subunits^6^. However, structural evidence of the direct interaction between Gα and ion channels remains elusive.

Here, we investigated the direct interaction between Gα and the transient receptor potential canonical 5 (TRPC5) ion channel in an appropriate functional context. Among a total of 6 subfamilies (TRPA, TRPC, TRPM, TRPML, TRPP, TRPV) of the TRP superfamily, TRPC channels share the highest sequence similarity to the prototypical *Drosophila melanogaster* TRP channel^7^. Based on their amino acid sequence similarity and response to diacylglycerol, seven TRPC channel members are divided into two groups, namely, TRPC1/4/5 and TRPC3/6/7. Of note, TRPC channels hold a canonical role in the phylogenetic tree as well; sequence similarities and recent accumulation of structural evidence indicate that all other TRP channels evolutionally diverge from TRPC channels^8–11^. Like many other TRP channels, TRPC5 is permeable to Ca^2+^, with little selectivity among monovalent cations^10^, and functions as a thermosensing channel^12,13^.

It has long been suggested that an alpha subunit of inhibitory G-protein (Gα_i_) can directly activate TRPC channels. Over the last 20 years, a considerable number of studies have been carried out in gastrointestinal (GI) tract and overexpression systems, and the findings have supported a functional relationship between Gα_i_ and TRPC4 or 5 channel^14–17^. In fact, the finding in GI smooth muscle cells that Gα_i_ protein could open the TRPC channel, induce depolarization, and increase membrane excitability shed a contrast against the general notion of Gα_i_ proteins that the activation of G_i_-coupled GPCRs elicits an efflux of potassium ions, hence hyperpolarizing the cells and decreasing overall membrane excitability, especially in cardiac nodal cells^18,19^. However, for all classes of channels asserted to be directly activated by Gα proteins, including TRPC channels, no complete structural evidence elaborating how Gα proteins directly regulate the channel activities have been identified.

Here, we present structures of the human TRPC5-Gα_i3_ protein complexes using cryo-electron microscopy (cryo-EM) and demonstrate that Gα_i3_ can directly activate TRPC5, increasing its sensitivity to phosphatidylinositol 4,5-bisphosphate (PIP_2_) using electrophysiology. Our results suggest that ion channels could be proclaimed as one of the direct effector molecules of Gα proteins.

## Results

### **Structure determination of the TRPC5-Gα**_**i3**_**complex**

For single-particle cryo-EM analysis and electrophysiological recordings, we have used a biochemically stable construct of the human TRPC5 channel in which the C-terminal loop region (residues 766–973) was truncated, and hereafter, we will call the construct TRPC5_EM_ in comparison to full-length TRPC5 channels (TRPC5_FL_). In HEK293T cells, overexpressed TRPC5_EM_ showed an intrinsic activity similar to that of TRPC5_FL_ channels (Supplementary Fig. 1a). Moreover, both channels showed similar increases in whole-cell current in response to the stimulation of coexpressed G_q_-coupled receptors (muscarinic acetylcholine receptor subtype 3, mAChR_3_) or G_i_-coupled receptors (mu-opioid receptor, μ-OR), or treatment with a specific and potent TRPC4 or 5 channel activator [(−)-Englerin A]^20^ (Supplementary Fig. 1b–d). In an attempt to mimic the action of G_i_-coupled receptor signaling, we also coexpressed constitutively active form of the Gα_i3_ protein, Gα_i3_^Q204L^. The glutamine-to-leucine mutation impairs the intrinsic GTPase activity of the Gα_i3_ protein, hence locking the protein in an active conformation once GDP-to-GTP exchange is accomplished^21^. When Gα_i3_^Q204L^ protein was coexpressed, both TRPC5_EM_ and TRPC5_FL_ showed significant increases in whole-cell current (Supplementary Fig. 1e). Both TRPC5_EM_ and TRPC5_FL_ showed a similar expression pattern in 3 different cell types (Supplementary Fig. 1f). Overall, we emphasize that the removal of the disordered C-terminus did not appear to alter the functional properties of TRPC5 in any of the functional measurements we made.

**Fig. 1:**
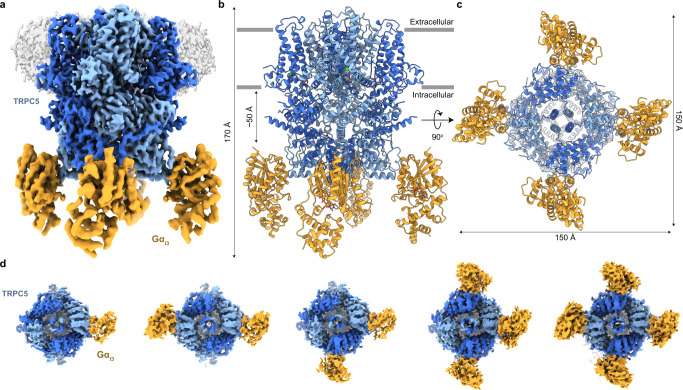
Overall architecture of the TRPC5-Gα_i3_ complex. **a** Composite map of the TRPC5-Gα_i3_ complex with four bound Gα_i3_ proteins. TRPC5 is colored in sky blue and dark blue by two subunits in the opposite tetrameric assembly. Gα_i3_ is colored yellow. The density for the nanodisc is shown in gray. Side view (**b**) and bottom view (**c**) of the atomic model of TRPC5-Gα_i3_. **d** Bottom views of the cryo-EM densities of the TRPC5-Gα_i3_ with various stoichiometries. All possible binding cases were observed: one, two in *trans*, two in *cis*, three, and four Gα_i3_ proteins (left to right) on a single TRPC5 channel.

We revealed the molecular architecture of the TRPC5-Gα_i3_ complex by using cryo-EM (Fig. 1a–c). Efforts to purify a stable TRPC5-Gα_i3_ complex in detergent solutions were unsuccessful. Therefore, we attempted to introduce several strategies for proper TRPC5-Gα_i3_ complex formation in vitro. First, we used the human Gα_i3_^Q204L^ construct, and recombinant Gα_i3_^Q204L^ was incubated with GTP prior to complexation with TRPC5 to maintain the constitutively active conformation of Gα_i3_^22^. Second, we coexpressed the Gα_i3_^Q204L^ mutant with yeast N-myristoyltransferase I to introduce myristoylation at the N-terminal glycine in the Gα_i3_^Q204L^ mutant^23^. Third, to facilitate membrane-associated interactions of the myristoylated Gα_i3_^Q204L^ with the channel, we reconstituted purified TRPC5 into lipid nanodiscs^24^. As a result, we successfully obtained the TRPC5-Gα_i3_ complex in lipid nanodiscs, a complex that comigrates stably in gel filtration (Supplementary Fig. 2a–i).

The initial 3D reconstruction of the TRPC5-Gα_i3_ complex yielded a 3.05 Å map with C4 symmetry imposition. Extra protein-like densities were other than the TRPC5 channel core, which turned out to be densities for Gα_i3_, were observed at the bottom of the channel. Compared to the TRPC5 channel core, the local resolution of Gα_i3_ densities was lower. We reasoned that the lower local resolution was due to not only inherent flexibilities but also various binding stoichiometries of the bound Gα_i3_ proteins (Supplementary Fig. 3a, b). To resolve the subpopulations according to the numbers of bound Gα_i3_ proteins, we performed 3D classification and particle sorting. As a result, 33.8% of the TRPC5 channels had one Gα_i3_ protein; 8.6% and 29.1% had two Gα_i3_ proteins in *trans*- and *cis*-conformation respectively; 22.3% had three Gα_i3_ proteins; and 6.2% had four Gα_i3_ proteins (Supplementary Fig. 3b). 3D reconstructions with C1 symmetry for each case resulted in density maps with corresponding numbers and conformations of Gα_i3_ proteins with improved densities for Gα_i3_ compared with the one before 3D classification and particle sorting, resulting in resolutions ranging from 3.6 to 4.0 Å (Fig. 1d and Supplementary Fig. 3b). Among those, the complex with four Gα_i3_ proteins showed the highest resolution of 3.54 Å when processed with C4 symmetry (Supplementary Fig. 3b, c). To improve the local resolution at the interface between TRPC5 and Gα_i3_, we further performed local refinement focused on the Gα_i3_ protein and its proximal binding region, which yielded a 4.19 Å focused map (Supplementary Fig. 3b, c). Finally, these maps were combined to make a composite map yielding a full complex model of the TRPC5-Gα_i3_ complex (Fig. 1a–c, Supplementary Fig. 3b, and Supplementary Table 1).

### **Overall architecture of the TRPC5-Gα**_**i3**_**complex**

The TRPC5-Gα_i3_ complex structure has dimensions of 150 Å × 150 Å × 170 Å (Fig. 1a–c). Each protomer of TRPC5 can bind one Gα_i3_ protein; therefore, a maximum of four Gα_i3_ subunits can be occupied in a tetrameric TRPC5. In our structure, densities for all structural domains for TRPC5 and Gα_i3_ were clearly observable, i.e., the ankyrin repeat domain (ARD), helix-loop-helix domain, transmembrane domain, connecting helix (CH) and coiled-coil domain (CCD) for TRPC5, and Ras-like domain and helical domain for Gα_i3_, as well as cations such as calcium and zinc ions in the previously known sites^25,26^ (Supplementary Fig. 4a, c). The overall architecture of TRPC5 in the TRPC5-Gα_i3_ complex was similar to the previously reported structures of tetrameric TRPC5 showing closed state^25–27^. Remarkably, the Gα_i3_ proteins bound to the ankyrin repeat edge of the channel, which was ~50 Å below the inner borderline of the lipid nanodiscs (Fig. 1a–c), and the solvent-accessible contact surface between TRPC5 and Gα_i3_ was approximately 690 Å^2^. The contact surface was formed by the insertion of ARD 1–2 into the cavity formed by α_2_ and α_3_ helices of Gα_i3_ (Fig. 1a, b and Supplementary Fig. 2a, b). The conformation of Gα_i3_ in our structure showed a typical GTP-bound form, i.e., the α_2_ helix was well-ordered and adopted a parallel orientation to the α_3_ helix, which was also supported by the distinct GTP-like density at the nucleotide-binding site of Gα_i3_ (Supplementary Fig. 4c).

Though we could not observe the density for the Gα_i3_-membrane interaction, we found that the N-myristoylation of Gα_i3_ was an indispensable element to accomplish a stable TRPC5-Gα_i3_ binding (Supplementary Fig. 2e–i). Since it was shown from a previous study that the αN helix in the inactive Gα was unfolded once the protein became active from GDP-to-GTP exchange^28^, the N-terminus of GTP-bound Gα_i3_ in this study could be unfolded from the Q204L mutation and the stable GTP incorporation (Supplementary Fig. 4c). The unfolded N-terminus then could span enough distance from the membrane such that Gα_i3_ could successfully position itself to the ankyrin repeat edge of the TRPC5 channel while the N-myristoylated moiety kept the Gα_i3_ being anchored to the membrane.

### **Activation of TRPC5 by Ca**^**2+**^**, PIP**_**2**,_**and Gα**_**i3**_

Although a number of studies have suggested that Gα_i_ proteins could activate the TRPC4 or 5 channels, most of them were either heavily dependent on whole-cell recordings^16,17,29^ or limited to endogenous currents in native tissues^15^, which is inherently short of a molecular level of specificity. To further investigate the direct activation of the channels by Gα_i3_, we measured the activity of TRPC5_EM_ in an excised inside-out patch using purified Gα_i3_ proteins.

**Fig. 2:**
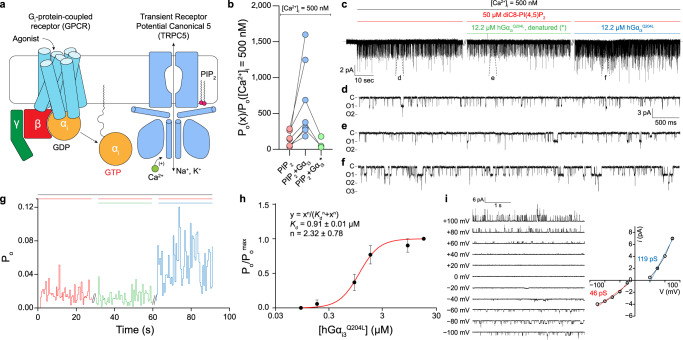
Functional evidence for direct activation of the TRPC5 channel by Gα_i3_. **a** Schematic drawing of a G_i_ protein-coupled receptor (G_i_PCR) activation coupled to TRPC5 channels. Once activated by agonizts, GPCR permits the Gα_i_ to exchange its nucleotide from GDP to GTP. The nucleotide exchange immediately releases Gα_i_ from Gαβγ. The GTP-bound Gα_i_ then interacts with effector molecules. Given that [Ca^2+^]_i_ is in the submicromolar range and that a physiological amount of phosphatidylinositol 4,5-bisphosphate (PIP_2_) is provided near the channel, the binding of Gα_i_ directly activates the TRPC5 channels. **b** Fold increase in the open probability (*P*_o_) with respect to each intracellular determinant (PIP_2_, PIP_2_ + Gα_i3_^Q204L^, or PIP_2_ + denatured Gα_i3_^Q204L^*). The mean open probability at each intracellular condition was divided by the mean open probability at which the patch was exposed to bath solution whose calcium concentration was buffered to 500 nM ([Ca^2+^]_i_ = 500 nM bath solution, see *Methods*). **c** Representative current trace for excised inside/out recordings of TRPC5 channels. The intracellular side of the same patch was exposed to either PIP_2_, PIP_2_ + Gα_i3_^Q204L^, or PIP_2_ + denatured Gα_i3_^Q204L^*. Slashes indicate 3 min of wash-out using [Ca^2+^]_I_ = 500 nM bath solution. **d–f** Current traces with an expanded time scale under corresponding conditions. **g** Open probability trace with respect to each intracellular condition. Colored bars above the trace indicate the corresponding intracellular conditions as in (**c**). **h** Dose-dependent action of Gα_i3_ proteins. Normalized open probability (*P*_o_/*P*_o_^max^) from six independent recordings was fitted into Hill’s equation. *K*_d_ and *n* represent the apparent dissociation constant and Hill coefficient, respectively. Symbols and bars represent the mean ± s.e.m. **i** (left) Current traces at different membrane potentials. Unitary currents (*i*) at 0 mV and +20 mV were indistinguishable from the baseline. (right) The *i*–V curve showed a doubly-rectifying shape. Slope conductances at both negative driving force and positive driving force were calculated from least-squared fit to linear equation. Symbols and bars represent the mean ± s.e.m. (*n* = 3).

Since a submicromolar level of intracellular calcium was necessary for receptor-operated activation of TRPC5 channels to be made^30^, we exposed the intracellular side of the patch to a bath solution in which the free-calcium ([Ca^2+^]_i_) concentration was buffered to 500 nM, unless otherwise mentioned. Although we were able to observe some but very scarce open events in the bath solution ([Ca^2+^]_i_ = 500 nM), the open probability of the TRPC5_EM_ channel was dramatically increased as soon as 50 μM diC8-PI(4,5)P_2_ was applied to the intracellular side of the patch (Fig. 2b–g). The results indicate that PIP_2_ could act as a cofactor at the intracellular leaflet in the process of channel activation, which is also in line with previous results^31^.

Notably, the open probability measured in the same excised patch was further increased when the patch was exposed to purified Gα_i3_^Q204L^ protein and PIP_2_ (Fig. 2b–g). The robust increase in open probability by Gα_i3_ was abolished when Gα_i3_ was heat denatured, suggesting that the activating effect is highly Gα_i3_^Q204L^-specific and not due to any other components in the bath solution (Fig. 2b–g).

The agonistic action of the Gα_i3_ protein was further examined by dose‒response experiments in an excised inside-out patch. The concentration of Gα_i3_ was sequentially increased from 0.07 to 14.6 μM in the presence of both calcium and PIP_2_, while the open probability was continuously examined in the same patch. The results showed that the apparent dissociation constant (*K*_d_) between Gα_i3_ and the TRPC5_EM_ channel was 0.91 μM. Moreover, fitting the result into the Hill equation yielded a Hill coefficient (n) of ~2.3, suggesting that the binding action of Gα_i3_ onto TRPC5 could be cooperative (Fig. 2h and Supplementary Fig. 5a, b). Notably, with the same concentration of PIP_2_ and Gα_i3_ protein, the activating effect of the two was severely diminished when [Ca^2+^]_i_ was clamped to near zero using 10 mM EGTA (Supplementary Fig. 5c–e), indicating that intracellular calcium is also a necessary cofactor for Gα_i3_-mediated channel activation.

The unitary currents of the TRPC5_EM_ channels from the excised patch at different membrane potentials yielded a unitary current-voltage (*i-*V) curve (Fig. 2i) whose shape is in line with the *i*-V curves from previous results^30,32,33^, suggesting that the currents measured from the inside/out patches were from the TRPC5 channels but not from other nonspecific channels. These results indicate that Gα_i3_ could directly activate the TRPC5 channels, and both calcium and PIP_2_ are necessary cofactors for Gα_i3_ to fully activate the channel.

### Two different conformations of TRPC5 and TRPC5-Gα_i3_

**Fig. 3:**
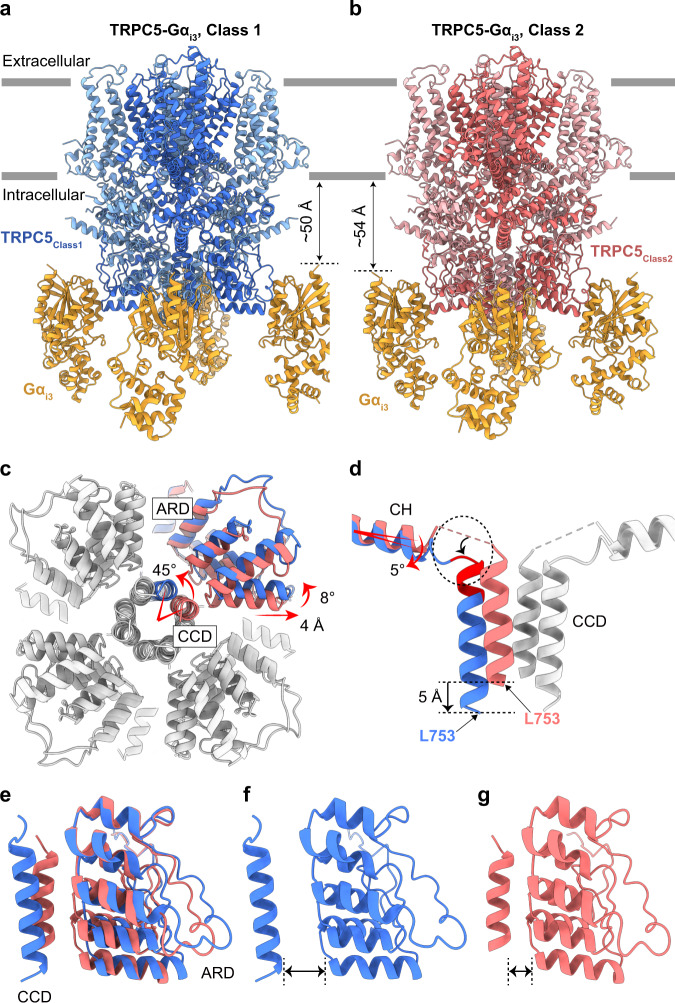
Two different conformations of the TRPC5-Gα_i3_ complexes. Atomic models of TRPC5_Class1_-Gα_i3_ (**a**) and TRPC5_Class2_-Gα_i3_ (**b**) viewed from the side. Conformational changes in the coiled-coil domain [(**c**) and (**d**)], ankyrin repeat domain (**c**) and the connecting helix (**d**) between TRPC5_Class1_-Gα_i3_ and TRPC5_Class2_-Gα_i3_. The superimposed models were viewed from the bottom (**c**) and the side (**d**). Gα_i3_ subunits are omitted for clarification. ARD, ankyrin repeat domain; CCD, coiled-coil domain; CH, connecting helix. Cartoon views of CCD and ARD of TRPC5_Class1_-Gα_i3_ (**f**), TRPC5_Class2_-Gα_i3_ (**g**), and their superposition (**e**).

Even after extensive 3D classifications and particle sorting according to the numbers of bound Gα_i3_ proteins, the four molecules of Gα_i3_ from the fully occupied TRPC5-Gα_i3_ complex (hereafter, TRPC5_Class1_-Gα_i3_) showed lower local resolution (Supplementary Fig. 3b). Moreover, when we performed 3D variability analysis of fully occupied TRPC5-Gα_i3_, we observed substantial movement of Gα_i3_ proteins and cytosolic domains of TRPC5, showing structural heterogeneity (Supplementary Movie 1). Therefore, to deal with this structural heterogeneity of the TRPC5_Class1_-Gα_i3_ complex, we performed focused 3D classification and sorted out approximately 30% of the TRPC5-Gα_i3_ particles with different conformations in the TRPC5 region (hereafter, TRPC5_Class2_-Gα_i3_) from TRPC5_Class1_-Gα_i3_ (Fig. 3a, b and Supplementary Fig. 3b). The entire TRPC5 region of TRPC5_Class2_-Gα_i3_ was almost identical to the TRPC5 structure in detergent (PDB code: 7E4T) with an RMSD of 0.557 Å (Supplementary Fig. 7b, g). In contrast, we observed substantial structural changes in the cytosolic domains, including the CCD and the ARD, when TRPC5_Class2_-Gα_i3_ was superimposed with TRPC5_Class1_-Gα_i3_ (Fig. 3c–g and Supplementary Fig. 7c, h). First, when viewed at the bottom of the channel, the CCD was rotated by 45° in a counterclockwise direction in TRPC5_Class1_-Gα_i3_ (Fig. 3c). This rotation was accompanied by the ordering and shortening of the loop between the CH and the CCD and the rotation of the connecting helix for 5°, thereby elongating the length of the CCD by a single helical turn and moving the CCD downward for 5 Å away from the membrane (Fig. 3d). Second, the ARD was also rotated by 8° in the same direction along with the CCD (Fig. 3c). Third, the ARD was stretched out from the symmetry axis by 4 Å, losing its interaction with the CCD (Fig. 3c, e–g). Fourth, we could newly observe a strong metal-like density surrounded by four His735 residues at the top of the CCD, which may have contributed to the stabilization of the rotated CCD (Supplementary Fig. 7k–n). Finally, despite all the differences mentioned above, Gα_i3_ was bound to both conformations of TRPC5 with different relative locations; that is, Gα_i3_ in the TRPC5_Class2_-Gα_i3_ complex showed a slightly longer distance (~4 Å) from the membrane and was located closer to the symmetry axis (Fig. 3a, b and Supplementary Fig. 7c).

To elucidate the structural features of TRPC5 prior to Gα_i3_ binding, we solved the cryo-EM structure of TRPC5 in the absence of Gα_i3_ in lipid nanodiscs, as all the previously reported structures of TRPC5 were in either detergent micelles or amphipols^25–27^. As a result, the obtained cryo-EM data yielded two structures: TRPC5_Class1_ at 3.15 Å and TRPC5_Class2_ at 3.59 Å, with a major distribution for the former (65%) and a minor distribution for the latter (35%) (Supplementary Fig. 6a–d and Supplementary Movie 2). Moreover, the structural comparisons of 1) TRPC5_Class1_ vs. TRPC5_Class1_-Gα_i3_ and 2) TRPC5_Class2_ vs. TRPC5_Class2_-Gα_i3_ showed almost identical conformations in the TRPC5 region, with RMSD of 0.514 Å and 0.456 Å, respectively (Supplementary Fig. 7d, e, i, j). Considering the similar particle distribution and structures of the two different conformations of TRPC5 in our structures of TRPC5 alone and TRPC5-Gα_i3_ complexes, we concluded that TRPC5 can exist in two different conformations regardless of Gα_i3_ binding.

### **Molecular determinants of Gα**_**i3**_**binding**

**Fig. 4:**
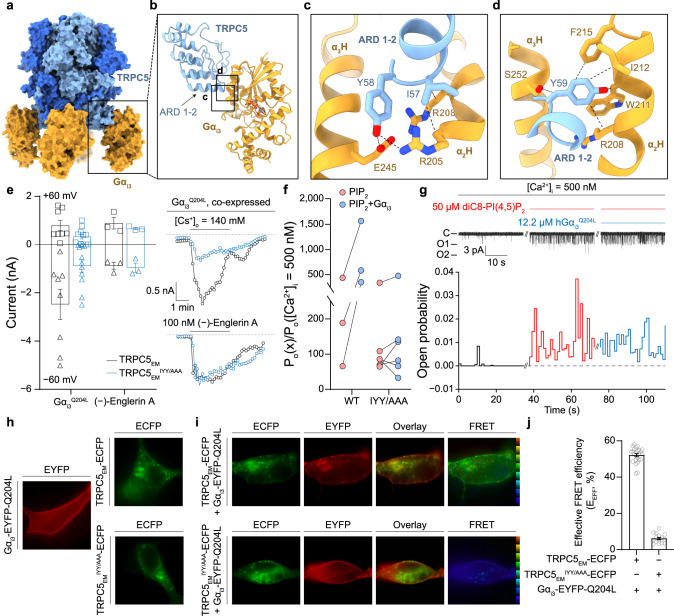
Molecular determinants of Gα_i3_ binding. **a** Surface representation of the TRPC5-Gα_i3_ complex. **b** Side view of an atomic model focused on the ankyrin repeat domain of TRPC5 and Gα_i3_. Boxes indicate the TRPC5-Gα_i3_ interfaces expanded in (**c**) and (**d**). **c**, **d** Interfaces between TRPC5 and Gα_i3_ with residues involved in TRPC5-Gα_i3_ binding. ARD, ankyrin repeat domain; α_2_H, α_2_ helix; α_3_H, α_3_ helix. **e** (Left) Summarized current amplitudes from both wild-type TRPC5_EM_ and mutant TRPC5_EM_^IYY/AAA^. When the IYY motif was changed to three consecutive alanine residues, the coexpression of Gα_i3_ could not render significant activation of the channel (*n* = 11), whereas wild-type channels were readily activated (*n* = 8). Both channels were similarly activated by 100 nM extracellular (−)-Englerin A, a highly potent and specific TRPC4 or 5 channel activator^20^. Squares and triangles represent whole-cell current at +60 mV and −60 mV, respectively. (Right) Representative current traces are also shown. **f** In an excised patch from cells expressing TRPC5_EM_^IYY/AAA^ channels, exposure of Gα_i3_ protein could not induce a significant increase in open probability of the channel. The response to PIP_2_, however, was similar in both wild-type and mutant channels (*n* = 3, wild-type; *n* = 6, mutant). **g** Representative current trace from the excised inside/out patch (top) and corresponding open probability trace (bottom). **h** Fluorescence signals from Gα_i3_^Q204L^ (EYFP), wild-type TRPC5 channel (TRPC5_EM_, ECFP) or a mutant channel (TRPC5_EM_^IYY/AAA^, ECFP). EYFP was fused between Ala114 and Glu115 of the Gα_i3_ protein, and ECFP was tagged on the C-termini of TRPC5 channels. **i** Epifluorescence images, overlay images, and FRET images from each expression pair. Colored E_EFF_ indicators on the right side of FRET images were set to cover from 0% (dark blue) to 100% (red) linearly. *Ex* excitation, *Em* emission. **j** Summary of the E_EFF_ from each expression pair (*n* = 24, wild-type; *n* = 15, mutant). Bars represent the mean ± s.e.m.

Although there are discrepancies in the relative location of Gα_i3_ bound to TRPC5 among the two different conformations of TRPC5 in TRPC5-Gα_i3_ complexes, it seems clear that Gα_i3_ shares an identical binding interface (Fig. 3a, b and Supplementary Fig. 7c). Furthermore, all-atom molecular dynamics (MD) simulation for the TRPC5-Gα_i3_ system showed that Gα_i3_ stably bound to the ARD throughout the 600-ns simulations (Supplementary Fig. 9a and Supplementary Movie 3). Therefore, we further examined the binding interface between Gα_i3_ and the ARD of TRPC5, which is formed by the insertion of the ARD 1–2 of TRPC5 into the groove formed by the α_2_ and α_3_ helices of Gα_i3_ (Fig. 4a, b). We observed that three TRPC5 residues (Ile57, Tyr58, and Tyr59; hereafter the IYY motif) appeared to contribute significantly to the binding (Fig. 4c, d). TRPC5 Ile57 interacted with Gα_i3_ Arg205 and Arg208, while Tyr58 participated in the hydrogen bonding network formed by Gα_i3_ Arg205 and Glu245. Furthermore, Tyr59 resided in close proximity to the hydrophobic residues belonging to the α_2_ and α_3_ helices of Gα_i3_, especially to the α_2_ helix (Trp211, Ile212, and Phe215). It is worth noting that the α_2_ and α_3_ helices of Gα are also used for interactions with PLCβ (Gα_q_), adenylyl cyclase (Gα_s_), phosphodiesterase (Gα_t_), and other effector molecules^34–36^. Another interesting finding of the IYY motif is that it was conserved in the corresponding region of TRPC4 (IYF instead of IYY) but not in any other TRPC subtypes (Supplementary Fig. 9b, c). Indeed, TRPC4 could be activated by the Gα_i_ series^15,29^, but TRPC1^29^ and 6 could not^16,17^. Overall, our findings suggest that the IYY motif is indeed crucial for the interaction between the TRPC5 and Gα_i3_ proteins.

In addition to the contribution of the IYY motif, the loop connecting the ARD 3-2 and 4-1 was also involved in the interaction between TRPC5 and Gα_i3_. The loop region seems to be highly flexible and has not yet been resolved in any of the previously determined TRPC5 structures^25–27^. However, we were able to observe clear densities corresponding to the loop, which may be stabilized through its interaction with the neighboring Gα_i3_ (Supplementary Figs. 4a, b, 10a, b). Moreover, electrostatic interactions between TRPC5 and Gα_i3_ also appeared to contribute to the binding (Supplementary Fig. 10c–i). Multiple acidic residues on the TRPC5 ankyrin repeat complemented an electropositive cavity on the binding surface of Gα_i3_ (Supplementary Fig. 10c–e). When the same area was exploited in TRPC6 channels, however, only a neutral surface potential was observable (Supplementary Fig. 10f). This finding offers another possible structural explanation for the observation that TRPC5 channels are activated by Gα_i_ proteins, but TRPC6 channels are not. Since electrostatic interactions can be manifested over a longer distance than van der Waals interactions, the attractive Coulomb force may guide the G-protein near TRPC5 channels so that two proteins can establish close contact using the IYY motif.

### Mutational analyses for the IYY motif

To evaluate the contributions of the interfacial residues to TRPC5-Gα_i3_ complex formation, the effects of mutating these residues were tested using electrophysiological recordings; i.e., we made a triple mutant TRPC5_EM_ channel in which the IYY motif was changed to three alanine residues (TRPC5_EM_^IYY/AAA^). The triple mutant channel showed a markedly reduced whole-cell current in response to the coexpression of Gα_i3_^Q204L^ compared to the wild-type channel (Fig. 4e). Among the three residues of the IYY motif, Tyr58 contributed the most to the binding (Supplementary Fig. 8). The response to (−)-Englerin A, however, was similar in both channels, suggesting that the mutation impaired the Gα_i3_-mediated activation process while leaving the pharmacological activation route intact. To further examine the role of the IYY motif in direct activation of the channel, we tested whether TRPC5^IYY/AAA^ could be activated by purified Gα_i3_^Q204L^ in the excised inside-out patch, as shown in Fig. 2. Consistent with whole-cell recordings, TRPC5^IYY/AAA^ could not be activated by Gα_i3_^Q204L^ (Fig. 4f, g). Even so, the open probability of TRPC5^IYY/AAA^ was still increased by intracellularly applied diC8-PIP_2_ (50 μM), as in wild-type channels (Fig. 4f, g). Overall, the results suggest that the IYY motif is exclusively utilized in the Gα_i3_-binding interface but is not involved in activation by other ligands, such as PIP_2_ or (−)-Englerin A.

The importance of the IYY motif was also confirmed by live-cell imaging experiments. When EYFP-fused Gα_i3_^Q204L^ (Gα_i3_-EYFP-Q204L) was expressed in HEK293T cells, a uniformly distributed fluorescence signal was observed across the entire plasma membrane, most likely indicating a membrane-anchored localization of the protein (Fig. 4h). When ECFP-fused TRPC5 (TRPC5_EM_-ECFP) was expressed, fluorescence signals were observed only in certain subsets of the plasma membrane (Fig. 4h). This punctate distribution of the channel was also similarly observed with the ECFP-fused TRPC5_EM_^IYY/AAA^ mutant channel (TRPC5_EM_^IYY/AAA^-ECFP) (Fig. 4h), suggesting that channel trafficking onto the plasma membrane is not impaired by the mutation.

The once membrane-delimited, uniformly distributed fluorescence signal of the Gα_i3_ protein, however, was dramatically changed as soon as the TRPC5 channels were coexpressed (Fig. 4i). Having abandoned the prior distribution, Gα_i3_ proteins almost identically colocalized with TRPC5 channels and followed the punctate distribution pattern of TRPC5 channels. This result suggests that Gα_i3_ proteins have a strong tendency to bind to TRPC5 channels, as long as the binding interface is exposed and not yet covered by other binding partners, such as Gβγ subunit, which in this study was most likely achieved through the constitutive binding of GTP inside the nucleotide-binding pocket (Q204L mutation) (Supplementary Fig. 4c). The Gα_i3_ protein and the TRPC5 channel showed evident colocalization; furthermore, strong Förster resonance energy transfer (FRET) was detectable (Fig. 4i, j). The effective FRET efficiency (E_EFF_) between the fluorophores on each protein—Gα_i3_ or TRPC5—was in a similar range to the E_EFF_ between an artificially linked ECFP-EYFP fluorophore pair (Supplementary Fig. 9h, i). Further calculation yielded that the apparent distance between the fused fluorophores was approximately 50 Å^37^. When Gα_i3_ proteins were coexpressed with the TRPC5^IYY/AAA^ mutant channel, however, Gα_i3_ not only maintained its original distribution pattern but also showed no noticeable colocalization with the channel (Fig. 4i). The FRET signal between TRPC5^IYY/AAA^-tagged ECFP and Gα_i3_-tagged EYFP was negligible and was comparable to the signal from randomly distributed ECFP and EYFP (ECFP + EYFP) (Fig. 4i, j).

To further confirm the contribution of the IYY motif to the Gα_i3_ interaction, the binding affinities between TRPC5_EM_ (or TRPC5_EM_^IYY/AAA^) and Gα_i3_^Q204L^ were examined by biolayer interferometry experiments (Supplementary Fig. 9d–f). As a result, the TRPC5_EM_ bound to Gα_i3_^Q204L^ with a dissociation constant (*K*_d_) of 0.53 μM (Supplementary Fig. 9d). In contrast, consistent with the electrophysiological recordings and live-cell imaging experiments, the IYY to AAA mutation nearly abolished binding to Gα_i3_^Q204L^ (Supplementary Fig. 9e). In a similar context, we also found that the once strongly co-immunoprecipitated TRPC5 and Gα_i3_ lost their in vivo interaction when the IYY/AAA mutation was introduced to TRPC5 channels (Supplementary Fig. 9i). Overall, the results of the electrophysiological, biochemical, and live-cell imaging analyses for the IYY motif indicated that the IYY motif of TRPC5 plays a critical role in Gα_i3_ binding to TRPC5 in a functional context.

### **Physiological role of Gα**_**i3**_**binding in light of PIP**_**2**_**-dependent regulation of TRPC5**

**Fig. 5:**
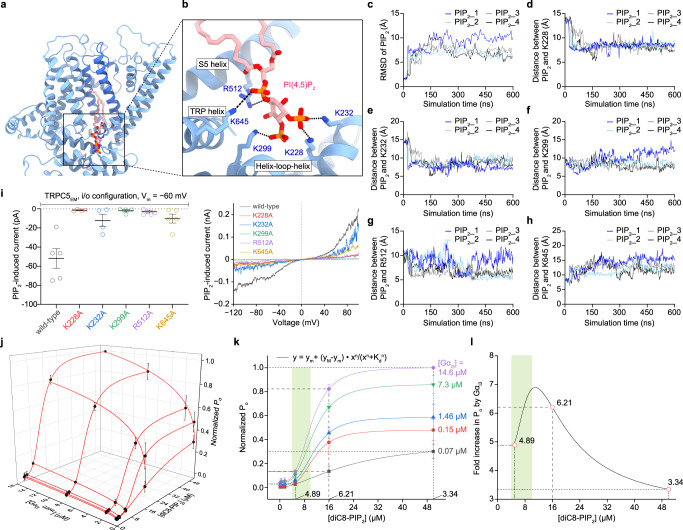
Putative PIP_2_ binding site and physiological role of Gα_i3_ in TRPC5 activation. **a** Representative structural snapshot of PIP_2_-bound TRPC5 by MD simulation. **b** Zoomed-in view of a putative PIP_2_ binding pocket in (**a**). **c** The RMSD of PIP_2_ plotted as a function of simulation time. The distance between center of mass of PIP_2_ inositol ring and center of mass of side chain of TRPC5 residues K228 (**d**), K232 (**e**), K299 (**f**), R512 (**g**), and K645 (**h**) plotted as a function of simulation time. **i** A summarized PIP_2_-induced current of wild-type and mutant channels (*n* = 5, wild-type; *n* = 4, K228A; *n* = 4, K232A; *n* = 4, K299A; *n* = 6, R512A; *n* = 6, K645A). Thick and thin lines represent the mean and s.e.m. *I*–*V* curves at current peaks from excised inside/out patches were also drawn. **j** Normalized open probability (*P*_o_) (black spheres and lines, mean ± s.e.m., *n* = 6–8 excised patches) is graphed as a function of Gα_i3_ and PIP_2_ concentrations. Lines were drawn from mathematical fit to the Hill equation. **k** Data points in (**j**) as a collection of curves (line projections onto the Normalized *P*_o_-[diC8-PIP_2_] plane) according to each Gα_i3_ concentration. Dotted lines and numbers at corresponding vertical intersections were drawn to explain how the fold increase in *P*_o_ by Gα_i3_ in (**l**) was obtained. **l** A Gα_i3_ amplification curve is shown as the purple curve ([Gα_i3_] = 14.6 μM) divided by the black curve ([Gα_i3_] = 0.07 μM) in (**k**). Three circles at [PIP_2_] = 5, 16, and 50 μM were calculated from the dotted lines in (**k**), but other data points in (**k**) and the curves encompassing them were deliberately omitted from the analysis to avoid the numerical singularity. In both (**k**) and (**l**), a tightly regulated physiological [PIP_2_] range^47^ is shown in green shadings.

Although the physical interaction between TRPC5 and Gα_i3_ and its effect on increasing the open probability of TRPC5 are evident from the results above, it appears that PIP_2_ is essential in TRPC5 activation (Fig. 2 and Supplementary Fig. 5). To obtain structural insights into PIP_2_ binding on TRPC5, we tried to incorporate PIP_2_ into TRPC5, but we were unsuccessful. As an alternative, we introduced MD simulations as computational microscopes. A binding site of PIP_2_ was hypothesized based on previous PIP_2_-dependent whole-cell recording results of TRPC4 and 5 channels^38^ and structural homology with PIP_2_-bound ion channels^39–45^. We predicted the binding pose using molecular docking and performed all-atom MD simulation of the PIP_2_-docked TRPC5 (see Methods). The PIP_2_ lipids bound stably at the putative binding site coordinated by 5 positively charged residues (K228, K299, K232, R512, and K645) throughout 600-ns simulations (Fig. 5a–h and Supplementary Movie 4). Furthermore, to validate the interactions between PIP_2_ and the surrounding residues of TRPC5, we performed mutational electrophysiological assays. Indeed, it showed that mutating any of the 5 positively charged residues significantly reduced the PIP_2_-induced currents of TRPC5 (Fig. 5i). On the basis of these findings, we propose that the PIP_2_ binding site on TRPC5 is located near the S2–S3 linker, S4–S5 linker, TRP helix, and helix-loop-helix region (Fig. 5a, b).

Although PIP_2_ is required for Gα_i3_ to modulate gating of the channel, it was clear that Gα_i3_ could form a complex with the channel even in the absence of PIP_2_ (Fig. 1). Therefore, we hypothesized that there could be some unknown thermodynamic coupling among the binding event of PIP_2_, the binding event of Gα_i3_, and the equilibrium between the open and closed states of the channel, namely, the open probability. Therefore, to investigate the potential interrelationship between PIP_2_ and Gα_i3_ in channel activation in detail, we measured the open probabilities of the channel with varying concentrations of PIP_2_ and Gα_i3_ (Fig. 5j). After exposure to intracellular solutions with differing concentrations of PIP_2_ and Gα_i3_, the patches were always exposed to the intracellular solution containing the maximum concentrations of both diC8-PIP_2_ (50 μM) and Gα_i3_^Q204L^ (14.6 μM), hence yielding the maximum open probability of the channels within the patch and therefore the normalized open probabilities.

**Fig. 6:**
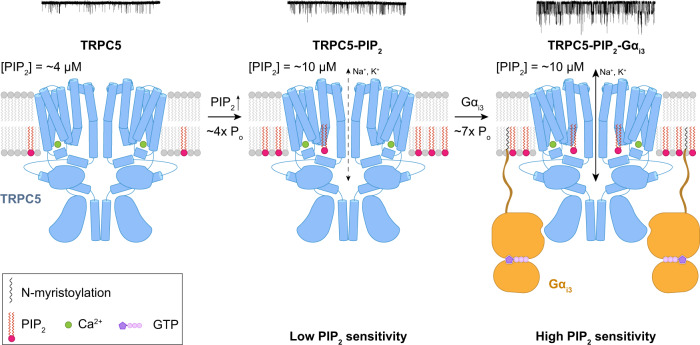
Possible mechanism for activation of the TRPC5 channels by Gα_i3_ and the cofactors Ca^2+^ and PIP_2_. Schematic drawing for a possible activation mechanism of TRPC5 channels by Ca^2+^, PIP_2_, and Gα_i3_. From electrophysiological recordings, we found that in the presence of intracellular Ca^2+^, the application of PIP_2_ at physiological concentrations (4–10 μM) onto the intracellular side of the patch increased the open probability of the channel four-fold on average. The binding of Gα_i3_ greatly increased the open probability approximately seven-fold with increased sensitivity of TRPC5 against PIP_2_. Representative current traces from the excised patch at each condition are attached. Only two diagonally opposed subunits of TRPC5, Gα_i3_, Ca^2+^ ions, and PIP_2_ molecules are shown for clarity.

As a result, we could obtain a three-dimensional dataset (Fig. 5j), and from the dataset, we compared the functional relationships between *P*_o_ and [diC8-PIP_2_] with respect to different [Gα_i3_] (Fig. 5k). First, when assessed with the Hill equation, we found that increasing the amount of Gα_i3_ lowered the apparent dissociation constant for PIP_2_ (*K*_d_ in Fig. 5k), hence increasing the PIP_2_ sensitivity of the channel. The dissociation constant for PIP_2_ (*K*_d_^PIP2^) of the TRPC5 channel was 23.3 ± 1.0 μM whereas the *K*_d_^PIP2^ of the TRPC5 with Gα_i3_ was 10.2 ± 0.1 μM. Second, from the given three-dimensional dataset (Fig. 5j), we tried to analyze how the *P*_o_-amplifying effect of Gα_i3_ proteins could vary along different PIP_2_ concentrations. Namely, we tried to solve an inverse problem so we could find a PIP_2_ domain where the amplifying effect of Gα_i3_ could be maximized. As a result, we found that the amplifying effect of Gα_i3_ showed a biphasic pattern with respect to [PIP_2_] (Fig. 5l). Within that biphasic pattern, we found that the highest amplifying effect of Gα_i3_ (5–7) could be found in the [PIP_2_] domain that could be briefly defined as 5 μM < [PIP_2_] < 12 μM (Fig. 5l). Interestingly, the deduced [PIP_2_] domain coincides with a tightly regulated, general physiological concentration of PIP_2_ in mammalian cells (4–10 μM)^46^. Therefore, the activation of Gα_i3_ proteins near the TRPC5 channel would result in approximately seven-fold increase in *P*_o_, provided that the PIP_2_ density in an inner leaflet of the plasma membrane remains physiological. On the basis of these findings, we propose that Gα_i3_ increases the sensitivity of TRPC5 to PIP_2_, thereby rendering TRPC5 more easily opened on the cell membrane, where the concentration of PIP_2_ is tightly regulated (Fig. 6).

## Discussion

What makes the TRP channel superfamily interesting is the wide spectrum of input modalities spanning a variety of physical and chemical stimuli by which the activity of the channels can be meticulously regulated^12,47–50^. In addition, we showed that TRPC5 channels permit Gα_i3_ proteins to directly regulate their activity. From the single-particle cryo-EM analysis, we not only presented a snapshot of the protein complex composed of the alpha subunit of G-protein and the ion channel, but with a comprehensive electrophysiological assay and all-atom MD simulation, we demonstrated crucial cofactors—Ca^2+^ and PIP_2_—for the proper activation of TRPC5 by the Gα_i_ protein. The analysis of the structure of the TRPC5-Gα_i3_ complex identified key binding residues, namely, the IYY motif. The functional correlation of the structural findings was also addressed in this study through electrophysiology, live-cell imaging, and biochemical assays.

In the case of the GIRK2 channel, the most prototypical model of the Gβγ-ion channel complex, PIP_2_ binding to GIRK2 is a prerequisite for Gβγ binding. The binding of PIP_2_ to the GIRK2 channel induces structural rearrangement of the cytoplasmic tail domain of the channel, which in concert generates the binding interface for Gβγ proteins^6,42^. In contrast, our current complex structure suggests that Gα_i3_ proteins could bind to TRPC5 even in the absence of PIP_2_, albeit PIP_2_ is required for Gα_i3_ proteins to gate the channel (Fig. 1).

Even though we found solid structural evidence that Gα_i3_ could bind to the channel without PIP_2_, in a functional aspect, it was crucial to maintain a sufficient amount of PIP_2_ to observe Gα_i3_ further promoting the opening of the channel. Namely, in the absence of PIP_2_, the effect of Gα_i3_ was almost totally nullified, which led us to conclude that the functional link between the binding event of Gα_i3_ and the actual favored opening of the channel is somehow uncoupled as long as the ambient [PIP_2_] near the channels is much lower than the *K*_d_^PIP2^ of the channel. Regarding the ambient PIP_2_ concentration in a native plasma membrane, it is generally conceived that physiological [PIP_2_] is tightly regulated in the midst of a dynamic equilibrium among PIP_2_-synthesizing or degrading enzymes. Even if the equilibrium greatly shifted toward a PIP_2_-degrading direction—such as episodic stimulation of the G_q/11_-phospholipase C (PLC) pathway or phosphoinositide 5-phosphatase –, a once deprived [PIP_2_] would always be replenished within tens of seconds to minutes^51^. That being said, it is highly unlikely to picture a general snapshot of the plasma membrane in which the ambient physiological concentration of PIP_2_ is unusually low, probably below a micromolar range. It could therefore be claimed that Gα_i3_ renders a more dynamic regulation of the channel activity provided that [PIP_2_] remains in the several micromolar range and that the concentration varies little as in a physiological scheme (Fig. 6). Even so, a potential caveat must be addressed that a soluble form of PIP_2_ (diC8-PIP_2_) instead of a natural form was used in all the experiments above. It is well known that natural PIP_2_ found in native plasma membrane with longer hydrophobic tails renders much stronger effect to binding partners compared to its soluble yet feasible isoforms. Therefore, we cannot assure how exactly the K_d_^PIP2^ reported in this study could precisely model the native circumstances in which natural PIP_2_ molecules interact with TRPC5 channels.

TRPC5 utilizing ARD as a binding interface was a truly thought-provoking finding. This is especially interesting because there are two other subfamilies in the TRP superfamily that have ARD in their structural architecture: TRPA and TRPV. Though the sequence homology is low, melastatin-homology-region (MHR) 3 and 4 of TRPM channels share a structural topology that is somewhat similar to the ARD of TRPC channels. The highly conserved binding interface of Gα proteins—α_2_ and α_3_ helices—for interactions with different kinds of effector molecules^34–36^ implies that a different set of channels may utilize different domains for binding to α_2_ and α_3_ of Gα proteins. Taken together, the structural confirmation given in this study may offer the groundwork for initiating further investigation of Gα protein modulation of a variety of ion channels.

## Methods

### Electrophysiology

Human embryonic kidney (HEK293-T) cells (ATCC) were maintained according to the supplier’s recommendations. For transient transfection, the cells were seeded in 12-well plates. The following day, plasmid was transfected using a transfection reagent X-tremeGENE 9 (Roche Molecular Biochemicals) as detailed in the manufacturer’s protocol. After 24–48 h, the cells were trypsinized and transferred to a small chamber on the stage of an inverted microscope (Eclipse Ti, Nikon). Whole-cell and single-channel currents were recorded at room temperature (18–20 °C) using borosilicate patch pipettes (Harvard Apparatus) of 2–3 MΩ and 15–25 MΩ, respectively. High pipette resistance for excised inside-out recording was achieved using a microforge (Narishige). The currents were recorded using an AxoPatch 200B patch-clamp amplifier (Molecular Devices). pClamp software v.10.7 or v.11.1 and Digidata 1440A (Molecular Devices) were used for data acquisition and application of the command pulses. Low-pass Bessel filter with 2 kHz cut-off frequency and 4 kHz sampling frequency were selected for whole-cell recording. For excised inside/out recording, 1 kHz low-pass Bessel filter cut-off frequency and 10 kHz sampling frequency were used.

Cancellation of pipette capacitance over 90% was achieved in cell-attached modes of every recording. After whole-cell configuration, almost total cancellation of whole-cell capacitance was achieved. Consequent manipulation of series resistance was necessary but the compensation of the series resistance was not achieved on purpose for access resistance remained significantly low (<3 MΩ) compared to membrane resistance at minimum (~30 MΩ). The data were analyzed using pClamp v.10.7 or v.11.1, Origin Pro 2016 software (OriginLab), and Prism 9 software (GraphPad).

The raw recording traces from excised inside/out patches were idealized using the 50% rule^52^ while signal changes in less than 0.16 ms (~1/2π*f*_*c*_) were ignored. All transitions were manually observed and verified. An open probability of a given condition was calculated in entire recording interval, typically ranging from 2 to 5 min. For data presentation, an arbitrary temporal window of 30 s was manually assigned within the entire recording interval. It was then always verified whether a mean open probability in the window is in similar range (95–105%) to the mean open probability of the entire recording interval. For visualization of the traces, digital Gaussian low-pass filter with cut-off frequency (*f*_*c*_) of 0.5 kHz was additionally applied without data reduction.

Open probability of the channel at given condition was analyzed as following. For a recording duration ${{{{{{\rm{T}}}}}}}_{{{{{{\rm{m}}}}}}}$ at *m*th condition, total number of open events (${{{{{{\rm{n}}}}}}}_{{{{{{\rm{o}}}}}}}$), closed events (${{{{{{\rm{n}}}}}}}_{{{{{{\rm{c}}}}}}}$), sum of each open dwell-time (${{{{{{\rm{\tau }}}}}}}_{{{{{{\rm{o}}}}}}}$) and each closed dwell-time (${{{{{{\rm{\tau }}}}}}}_{{{{{{\rm{c}}}}}}}$) were calculated respectively, from the idealized recording trace. Absolute current amplitudes for closed state and open states were assigned based on all-point histogram analysis. Namely,

1 $${{{{{{\rm{P}}}}}}}_{{{{{{\rm{o}}}}}}}=\frac{{\sum }_{i=1}^{{n}_{o}}{\tau }_{o,i}}{{{{{{{\rm{T}}}}}}}_{{{{{{\rm{m}}}}}}}}$$

For bath solutions for whole-cell recording of TRPC5 channels, we used Normal Tyrode solution unless otherwise mentioned. The Normal Tyrode solution contained 135 mM NaCl, 5 mM KCl, 2 mM CaCl_2_, 1 mM MgCl_2_, 10 mM Glucose, 10 mM HEPES with pH of 7.4 adjusted with NaOH. Cs-rich solution contained 140 mM CsCl, 2 mM CaCl_2_, 1 mM MgCl_2_, 10 mM Glucose, 10 mM HEPES with a pH of 7.4 adjusted with CsOH. The pipette solution for whole-cell recording contained 140 mM CsCl, 0.5 mM EGTA, 10 mM HEPES, 3 mM Mg-ATP, 0.2 mM Tris-GTP with a pH of 7.2 adjusted with CsOH.

The bath solutions for excised inside/out recording contained 140 mM L-Aspartic acid, 140 mM CsOH, 10 mM HEPES, 10 mM EGTA, 7.13 mM CaCl_2_ ([Ca^2+^]_i_^free^ = 500 nM), 3.31 mM MgCl_2_ ([Mg^2+^]_i_^free^ = 3 mM) with a pH of 7.2 using CsOH. The 10 EGTA solution contained 140 mM L-Aspartic acid, 140 mM CsOH, 10 mM HEPES, 10 mM EGTA, 4.06 mM MgCl_2_ ([Mg^2+^]_i_^free^ = 3 mM) with a pH of 7.2 using CsOH. Chelator-free concentrations of divalent cations were calculated from WebMaxC Extended Calculator (UC Davis Pharmacology)^53^. The pipette solution for excised inside/out recording contained 140 mM L-Aspartic acid, 140 mM CsOH, 10 mM HEPES, 10 mM Glucose, 2 mM CaCl_2_, 1 mM MgCl_2_ with a pH of 7.4 using CsOH.

For whole-cell recording, ramp pulse from −120 mV to +100 mV in 0.55 s was applied in every 10 s. Holding potential was maintained at −60 mV. For inside/out recording, a membrane potential (${{{{{{\rm{V}}}}}}}_{{{{{{\rm{mem}}}}}}}$) was calculated as following

2 $${{{{{{\rm{V}}}}}}}_{{{{{{\rm{mem}}}}}}}={{{{{{\rm{V}}}}}}}_{{{{{{\rm{rev}}}}}}}-{{{{{{\rm{V}}}}}}}_{{{{{{\rm{cmd}}}}}}}$$

where ${{{{{{\rm{V}}}}}}}_{{{{{{\rm{rev}}}}}}}$ indicates reversal potential of the channel at given intracellular/extracellular solution pair, and ${{{{{{\rm{V}}}}}}}_{{{{{{\rm{cmd}}}}}}}$ implies commanding potential applied from the AxoPatch 200B patch-clamp amplifier. Liquid junction potential (LJP) was calculated using pClamp 11.1 software and LJP less than ±3 mV was neglected from the calculation.

For the delivery of PIP_2_, G-protein, or PIP_2_ + G protein sample onto the intracellular side of the patch, an additional, left-handed micromanipulator (uMp, Sensapex) was mounted. Solutions were back-filled into the pipette and mounted on a pipette-holding accessory (PicoPump, World Precision Instrument). For the application of the materials inside the glass pipette, PicoPump unit was digitally controlled by DigiData 1440 and Clampex 11.1 software.

### In vitro **G-protein activation and/or PIP**_**2**_**preparation for inside/out patch-clamp recording**

Before the activation process, purified G-proteins aliquoted with a volume of *V*_aliq_ (ml) were flash-frozen using liquid nitrogen and stored at −80 °C. A bath solution of interest was selected as a Base Buffer (BB). Using 984 μl of BB, 1 μl of 0.2 M DTT, 5 μl of 0.2 M Tris-GTP, and 10 μl of 1% CHAPS (m/V) was diluted to make 1 ml of Final Buffer (FB). (1−*V*_aliq_) ml of FB was added onto pre-aliquoted purified G-protein and was incubated at 30 °C for 1 h for in vitro G-protein activation^54^. For buffer exchange, sample was loaded onto Amicon-Ultra-0.5 ml-10K (Merck Millipore) buffer exchange kit. After three times of wash-out using BB, sample was retrieved and incubated on ice. If further processes were not necessary, BB was added onto the retrieved sample so that final volume of the sample become 200 μl. *V*_aliq_ was calculated so that the protein concentration at 200 μl volume could be 0.5 mg ml^−1^ (12.2 μM).

diC8-PIP_2_ (0.5 mg) was purchased from Avanti Polar Lipids and diluted with BB so that the concentration of the solution is 250 μM. For PIP_2_-only condition, 160 μl of BB was added onto 40 μL of aliquoted PIP_2_ solution, so that final concentration of the PIP_2_ could be 50 μM. For PIP_2_ + G protein condition, 40 μl of aliquoted PIP_2_ was added onto the retrieved G-protein sample on the ice, and BB was further added so that total volume of the solution could be 200 μl. For the denaturation of the G-protein, 1 M DTT and 9 M Urea was added into the purified and aliquoted G-protein. Final concentration of DTT was 50 mM and urea was 6 M. The aliquot sample containing DTT, and urea was incubated 5 min in heat-block with the temperature of 95 °C.

### Co-immunoprecipitation (Co-IP) and immunoblotting

For immunoblotting, cells were seeded in six-well plates. On the next day, 0.5–2 μg of plasmid was transfected into cells using the transfection reagent Lipofectamine 2000 (Invitrogen) according to the manufacturer’s recommendation. After 24 h, the cells were harvested as following. Lysates were prepared in lysis buffer (0.5% Triton X-100, 50 mM HEPES, 120 mM NaCl, 2 mM EDTA, 2 mM MgCl_2_, pH 7.5 adjusted by NaOH) via passaging 10–15 times through a 26-gauge needle. After lysates were centrifuged at 13,000 × *g* for 1 min at 4 °C, the protein concentration in the supernatants was determined. The extracted proteins in a sample buffer were loaded onto 10% Tris-glycine sodium dodecyl sulfate-polyacrylamide gel electrophoresis (SDS‒PAGE) gels. The proteins were transferred onto nitrocellulose membrane.

For co-immunoprecipitation, 500 μl of cell lysates (500–1000 μg) were incubated with 1 μg of anti-Flag or anti-Gα_i3_ antibodies and 30 μl of protein G-agarose beads at 4 °C overnight with gentle rotation. After beads were washed three times with wash buffer (same as lysate buffer except for 0.1% Triton X-10 instead of 0.5%), the precipitates were then eluted with 30 μl of 2× Laemmli buffer and subjected to immunoblotting.

For immunoblotting, antibodies were diluted as following: anti-Flag (1:3000), anti-Gα_i3_ (1:1000), anti-β-tubulin (1:3000). The ratio refers to volumetric ratio. For instance, 1 μl of anti-Flag antibody was diluted in 3 ml of buffer solution. Detailed information on antibodies is listed in the Reporting Summary.

### Live-cell image acquisition and Förster Resonance Energy Transfer (FRET) measurements

HEK293-T cells were cultured in a 35-mm coverslip bottom dish or a 12-well plate to obtain images and measure FRET efficiency. For the fluorophore pair, an enhanced cyan fluorescent protein (ECFP) was inserted at the C-terminus of human TRPC5 channels with an in-house linker sequence (5′-GCG TCG ACG GTA CCG CGG GCC CGG GAT CCA CCG GTC GCC ACC-3′), and an enhance yellow fluorescent protein (EYFP) was inserted in between alanine on 114th residue and glutamate on 115th residue, as previously mentioned^22,55,56^. The human TRPC5 plasmid in pEGFP-N1 vector was kindly donated by Dr. S. Kaneko, and human Gα_i3_^Q204L^ plasmid in pcDNA3.1(+) vector was purchased from cDNA Resource Center (Bloomsburg, PA, United States). All subcloning was conducted using Gibson Assembly^57^. To obtain the image and FRET efficiency of a cell, we used an inverted microscope (Ti, Nikon) with a 100× oil objective lens (S Fluor, Nikon) and the three cube FRET calculation^58^ controlled by MetaMorph 7.10 software (Molecular Devices). The three-cube FRET efficiency (cube settings for CFP, YFP, and Raw FRET) was acquired from a LED light source (pE-4000, CoolLED) to three-cube FRET (excitation, dichroic mirror, filter) through a fixed collimator: CFP/YFP FRET (ET 435/20 nm, ET CFP/YFP/mCherry beam splitter, ET 535/30 nm, Chroma). After locating cell of interest in a visual field, excitation wavelengths for CFP and YFP were applied sequentially. The elapsed time for the exchange of excitation wavelengths was usually less than 200 ms and was almost exclusively affected by an exposure time but not by a control speed limit of the light source (<0.5 ms). Emission signals from CFP and YFP fluorophores were selectively acquired through a dual-view beam splitter (DV2, Teledyne Photometrics). Split images were acquired on a cooled 3 MHz (14 bit) sCMOS (optiMOS) camera (Teledyne Photometrics) with an exposure time of 100–200 ms without binning under control of MetaMorph 7.10 software. FRET signal was measured from a cell only if relative intensities of both fluorophores (CFP and YFP) are in similar range (0.5 < *I*_CFP_/*I*_YFP_ < 2.0). Acquired images were aligned using an in-house program written in MatLab (Mathworks, USA). MetaFluor 7.10 software was used for FRET-gradient representation of the images.

### Computation of FRET Ratio (FR) and Effective FRET efficiency (E_EFF_)

The FRET Ratio (FR)^58^ is equal to the fractional increase in YFP emission due to FRET and was calculated as following.

3 $${{{{{\rm{FR}}}}}}=\frac{{{{{{{\rm{F}}}}}}}_{{{{{{\rm{AD}}}}}}}}{{{{{{{\rm{F}}}}}}}_{{{{{{\rm{A}}}}}}}}=\frac{{{{{{{\rm{S}}}}}}}_{{{{{{\rm{FRET}}}}}}}\left({{{{{\rm{DA}}}}}}\right)-{{{{{{\rm{R}}}}}}}_{{{{{{\rm{D}}}}}}1}\cdot {{{{{{\rm{S}}}}}}}_{{{{{{\rm{CFP}}}}}}}\left({{{{{\rm{DA}}}}}}\right)}{\left({{{{{{\rm{R}}}}}}}_{{{{{{\rm{A}}}}}}1}\cdot \left[{{{{{{\rm{S}}}}}}}_{{{{{{\rm{YFP}}}}}}}\left({{{{{\rm{DA}}}}}}\right)-{{{{{{\rm{R}}}}}}}_{{{{{{\rm{D}}}}}}2}\cdot {{{{{{\rm{S}}}}}}}_{{{{{{\rm{CFP}}}}}}}\left({{{{{\rm{DA}}}}}}\right)\right]\right)}$$

Here, ${{{{{{\rm{S}}}}}}}_{{{{{{\rm{CUBE}}}}}}}\left({{{{{\rm{SPECIMEN}}}}}}\right)$ denotes an intensity measurement, where ${{{{{\rm{CUBE}}}}}}$ indicates the filter cube (CFP, YFP, or FRET), and ${{{{{\rm{SPECIMEN}}}}}}$ indicates whether the cell is expressing the donor (D; CFP), acceptor (A; YFP), of both (DA). The predetermined constants, namely,

4 $${{{{{{\rm{R}}}}}}}_{{{{{{\rm{D}}}}}}1}=\frac{{{{{{{\rm{S}}}}}}}_{{{{{{\rm{FRET}}}}}}}\left({{{{{\rm{D}}}}}}\right)}{{{{{{{\rm{S}}}}}}}_{{{{{{\rm{CFP}}}}}}}\left({{{{{\rm{D}}}}}}\right)},{{{{{{\rm{R}}}}}}}_{{{{{{\rm{D}}}}}}2}=\frac{{{{{{{\rm{S}}}}}}}_{{{{{{\rm{YFP}}}}}}}\left({{{{{\rm{D}}}}}}\right)}{{{{{{{\rm{S}}}}}}}_{{{{{{\rm{CFP}}}}}}}\left({{{{{\rm{D}}}}}}\right)},{{{{{{\rm{R}}}}}}}_{{{{{{\rm{A}}}}}}1}=\frac{{{{{{{\rm{S}}}}}}}_{{{{{{\rm{FRET}}}}}}}\left({{{{{\rm{A}}}}}}\right)}{{{{{{{\rm{S}}}}}}}_{{{{{{\rm{YFP}}}}}}}\left({{{{{\rm{A}}}}}}\right)}$$

are from measurements applied to single cells expressing only CFP- or YFP-tagged molecules. Although three-cube FRET does not require that CFP and YFP fusion constructs preserve the spectral features of the unattached fluorophores, similar ratios and recorded spectra furnished two indications that the spectral features of the fluorophores were largely unperturbed by fusion. Since FR relies on YFP emission, YFP should be attached to the presumed limiting moiety in a given interaction. Subsequent quantitative calculations based on FR relied on a presumed 1:1 interaction stoichiometry.

The effective FRET efficiency (${{{{{{\rm{E}}}}}}}_{{{{{{\rm{EFF}}}}}}}$) was determined by

5 $${{{{{{\rm{E}}}}}}}_{{{{{{\rm{EFF}}}}}}}={{{{{\rm{E}}}}}}\cdot {{{{{{\rm{A}}}}}}}_{{{{{{\rm{b}}}}}}}=\left({{{{{\rm{FR}}}}}}-1\right)\cdot \left[\frac{{{{{{{\rm{\epsilon }}}}}}}_{{{{{{\rm{YFP}}}}}}}\left(440\right)}{{{{{{{\rm{\epsilon }}}}}}}_{{{{{{\rm{CFP}}}}}}}\left(440\right)}\right]$$

where ${{{{{\rm{E}}}}}}$ is the intrinsic FRET efficiency when fluorophore-tagged molecules are associated with each other, ${{{{{{\rm{A}}}}}}}_{{{{{{\rm{b}}}}}}}$ is the fraction of YFP-tagged molecules that are associated with CFP-tagged molecules, and the bracketed term is the ratio of YFP and CFP molar extinction coefficients (${{{{{\rm{\epsilon }}}}}}$) scaled for the FRET cube excitation filter^59^. We determined this ratio to be 0.079 based on maximal extinction coefficients for ECFP and EYFP^60^, excitation spectra, and comparison to an in-house donor-dequenching FRET result as a positive control with a quality control reference ^61^. All computations were conducted using in-house program written in MatLab (Mathworks).

### Expression and purification of human TRPC5 in lipid nanodiscs

A gene fragment of truncated human TRPC5 (amino acids 1 to 765, TRPC5_EM_) was subcloned into an in-house-modified pEG-BacMam vector in frame with a C-terminal HRV-3C recognition site followed by EGFP and Twin-StrepII-tag. The protein was expressed in HEK293S GnTI^-^ (N-acetylglucosaminyltransferase I-negative, ATCC) suspension cells using the BacMam system. Baculovirus was generated using *Spodoptera frugiperda* (Sf9) insect cells and amplified according to the manufacturer’s protocol. P3 baculovirus was added to HEK293S GnTI^−^ suspension cells at a cell density of 2.0–2.5 M cells ml^−1^. After 12–24 h of incubation, 10 mM sodium butyrate was added to the cell culture, and the cells were further grown for 48–60 h at 30 °C before being harvested.

All purification steps were carried out at 4 °C or on ice unless indicated otherwise. The collected cells were resuspended in Buffer A (50 mM Tris-HCl, pH 8.0, 150 mM NaCl, and 1 mM DTT) supplemented with protease inhibitors [1 mM phenylmethylsulfonyl fluoride (PMSF), 2 μg ml^−1^ leupeptin, 2 μM pepstatin A, and 2 μM aprotinin] and solubilized with 1% (w/v) N-dodecyl-β-d-maltopyranoside (DDM), and 0.1% cholesteryl hemisuccinate (CHS) for 1 h. After ultracentrifugation at 100,000 × *g* for 1 h, the supernatant was incubated with Strep-Tactin resin (IBA Lifesciences) for 3–6 h. The resin was washed with 10 column volumes of Buffer B [Buffer A supplemented with 0.1% (w/v) digitonin and 0.01% (w/v) CHS]. The protein was eluted with two column volumes of Buffer B with 5 mM d-desthiobiotin.

Before nanodisc reconstitution, MSP2N2 was expressed and purified with minor modifications^62^. In brief, a plasmid containing MSP2N2 with a hexa-histidine tag was transformed into BL21(DE3) cells (Invitrogen). The cells were grown in Luria-Bertani (LB) medium and protein expression was induced by the addition of 0.5 mM isopropyl-β-D-1-thiogalactopyranoside (IPTG) when the optical density of the culture at 600 nm reached 0.6–1.0. Cells were harvested after 4 h of incubation at 18 °C. The cells were then resuspended in a buffer containing 100 mM Tris-HCl, pH 8.0, 100 mM NaCl, 10% glycerol, 1 mM PMSF, and 5 mM imidazole and disrupted with a microfluidizer (Microfluidics). After centrifugation at 25,000 × *g* for 30 min, MSP2N2 was purified by Ni-affinity chromatography, and the eluent was buffer-exchanged to Buffer A. Finally, the buffer-exchanged MSP2N2 was concentrated to 6–8 mg ml^−1^, flash-frozen and stored at −80 °C until use.

For nanodisc reconstitution, TRPC5 was mixed with in-house-purified MSP2N2 and soybean lipids at a molar ratio of TRPC5 monomer:MSP2N2:soybean lipids = 1:3:200. After removing the detergent by BioBead (Bio-Rad), the sample was buffer-exchanged to Buffer A using a PD-10 desalting column (Bio-Rad) to remove d-desthiobiotin and was subjected to 2nd StrepII affinity chromatography to remove excess MSP2N2 proteins. The elute was concentrated and loaded onto a Superose 6 10/300 GL column (Cytiva) pre-equilibrated with Buffer A for further purification, or flash-frozen and stored at −80 °C without further purification for future complex formation with Gα_i3_ proteins. For the purification of the TRPC5_EM_^IYY/AAA^ construct, the IYY/AAA mutation was introduced to the TRPC5_EM_ plasmid, and all the cell culture and purification procedures were identical to those of TRPC5_EM_.

### Expression and purification of N-myristoylated human Gα_i3_^Q204L^

N-myristoylated human Gα_i3_^Q204L^ was expressed and purified as described previously with some modifications^23,63^. In brief, human Gα_i3_ was subcloned into a pRSF vector (Novagen) with a point mutation Q204L, and a hexa-histidine tag was inserted between amino acid residues M119 and T120. Wild-type yeast N-myristoyltransferase I (NMT1) was subcloned into a pGST2 vector without any fusion protein or affinity tag. Both plasmids for Gα_i3_^Q204L^ and NMT1 were co-transformed in RosettaII (DE3) cells (Invitrogen) for protein expression. The cells were grown in Terrific Broth medium, and protein expression was induced by the addition of 0.1 mM IPTG when the optical density of the culture at 600 nm reached 0.6–1.0. Cells were harvested after overnight incubation at 18 °C.

All purification steps were carried out at 4 °C or on ice unless indicated otherwise. The cells were resuspended in a buffer containing 50 mM Tris-HCl, pH 8.0, 100 mM NaCl, 1 mM MgCl_2_, 10 μM GTP, 1 mM PMSF, and 5 mM imidazole and disrupted with a microfluidizer (Microfluidics). After centrifugation at 25,000 × *g* for 30 min, the Gα_i3_^Q204L^ was purified by Ni-affinity chromatography followed by hydrophobic interaction chromatography. Finally, the sample was further purified by size-exclusion chromatography using a Superdex200 10/300 GL column (Cytiva), pre-equilibrated with Buffer A with 5 μM GTP. Fractions containing N-myristoylated Gα_i3_^Q204L^ proteins were concentrated, flash-frozen, and stored at −80 °C until use. For the expression of N-myristoylated Gα_i3_^WT^, a plasmid containing Gα_i3_^WT^ with a hexa-histidine tag insertion but without Q204L mutation was co-transformed with NMT1. For the expression of non-myristoylated Gα_i3_^Q204L^, only a plasmid containing human Gα_i3_^Q204L^ was transformed in RosettaII (DE3) cells. All the cell culture and purification procedures were identical to those in the case of N-myristoylated Gα_i3_^Q204L^.

### **Complex formation of TRPC5-Gα**_**i3**_**in lipid nanodiscs**

Before complex formation, the purified N-myristoylated Gα_i3_^Q204L^ was activated by adding 1 mM GTP and incubated for 1 h at 30 °C. After removal of any precipitate by centrifugation, the GTP-activated N-myristoylated Gα_i3_^Q204L^ was mixed with nanodisc-reconstituted TRPC5 at a Gα: TRPC5 monomer = 6:1 molar ratio and incubated overnight at 4 °C. To remove the EGFP and Twin-StrepII-tag on TRPC5, HRV-3C protease was added and incubated for 1 h. Finally, the protein solution was centrifuged and loaded onto a Superose 6 10/300 GL column pre-equilibrated with Buffer A with 5 μM GTP for size-exclusion chromatography. Peak fractions were analyzed by SDS‒PAGE, and fractions containing TRPC5, MSP2N2, and Gα_i3_ were used for cryo-EM structure determination.

### Biolayer interferometry

Biolayer interferometry experiments were performed using a BLItz system (ForteBio) with amine-reactive 2nd generation biosensors (AR2G, ForteBio). Purified EGFP-tagged TRPC5 (TRPC5_EM_ or TRPC5_EM_^IYY/AAA^) in lipid nanodiscs (500 nM) was immobilized onto the biosensor in a buffer consisting of 20 mM HEPES-NaOH, pH 8.0, and 150 mM NaCl using NHS/EDC-mediated crosslinking. Purified N-myristoylated Gα_i3_^Q204L^ was activated with 1 mM GTP and then buffer-exchanged into the buffer consisting of 20 mM HEPES-NaOH, pH 8.0, and 150 mM NaCl. Next, the GTP-activated Gα_i3_^Q204L^ flowed with concentrations of 0.31 μM, 0.47 μM, 0.63 μM, 0.94 μM, and 1.25 μM for 300 s for association, and dissociation was measured for another 300 s. As a negative control, TRPC5_EM_ in lipid nanodiscs was used as analyte, and N-myristoylated Gα_i3_^WT^ was used as the ligand. In this case, all procedures were performed equally except that 1 mM GDP instead of GTP was used. The data were analyzed by “Specific binding with Hill slope” using GraphPad Prism 9.3.1 (GraphPad).

### Cryo-EM sample preparation and data acquisition

Purified TRPC5 and TRPC5-Gα_i3_ complex was concentrated to 2.5–3.5 mg ml^−1^. A final concentration of 2.5 mM of CaCl_2_ was added to the samples and incubated for 30 min before vitrification. The cryo-EM grids were prepared by applying 3 μl of protein sample to a glow-discharged Quantifoil 1.2/1.3 300-mesh Cu holey carbon grids (Electron Microscopy Sciences) under 4 °C and 100% humidity. The grids were blotted for 7.0 s and then plunged into liquid ethane using Vitrobot Mark IV (FEI).

Cryo-EM data collection was performed on a Glacios at 200 kV accelerating voltage in the Center for Macromolecular and Cell Imaging (Seoul National University). Movies were recorded using a Falcon 4 detector in counting mode using EPU 2.10 automated data-acquisition program. For the TRPC5-Gα_i3_ complex structure, 5,590 movies were collected with a pixel size of 1.088 Å/pixel using a defocus range of −1.0 to −2.0 μm. Each movie (40 frames) was acquired using a total exposure of 40 e^−^/Å^2^. For the TRPC5 structure in lipid nanodiscs, 5,523 movies were collected with a pixel size of 1.086 Å/pixel using a defocus range of −1.0 to −2.0 μm. Each movie (40 frames) was acquired using a total exposure of 40 e^−^/Å^2^.

### Cryo-EM data processing

For the TRPC5-Gα_i3_ complex, 5590 collected movies were subjected to motion correction and CTF estimation using patch motion correction and patch CTF in CryoSPARC version 3.2.0^64^. After several rounds of 2D classifications, 362,033 Gα_i3_ protein-bound particle images were selected and subjected to 3D reconstruction using ab initio reconstruction followed by non-uniform refinement with C4 symmetry imposed in CryoSPARC. The particles were then transferred to Relion version 4.0^65^ using the “csparc2star.py” command, and Bayesian particle polishing and CTF refinement were performed. Post-processing with shiny particles and 3D maps from CryoSPARC yielded a 3.05 Å consensus map, of which resolution was determined by gold-standard 0.143 Fourier shell correlation (GSFSC). To further classify the particles according to the numbers and conformations of bound Gα_i3_ proteins, we performed focused 3D classification and particle sorting processes. The particles used to obtain the consensus map were symmetry-expanded with C4 symmetry and further subjected to particle subtraction to generate subtracted particles with the density of one ARD-Gα_i3_ only. Further focused 3D classification without image alignment using the same soft mask used for the particle subtraction yielded two classes with strong densities for Gα_i3_ (class 1 and 2; Gα_i3_-bound classes) and another two classes with very weak densities for Gα_i3_ (class 3 and 4; Gα_i3_-unbound classes). Next, we traced the focused 3D classes back to their original particles and analyzed what kinds of classes compose each original particle by analyzing metadata using customized “awk” scripts. After sorting the original particles according to the possible cases of combinations of the classes using customized “awk” scripts and removing symmetry-expanded duplicates, we reverted to original particles and generated “particles.star” files according to the numbers and conformations of bound Gα_i3_ proteins. The sorted particle images were then transferred back to CryoSPARC and subjected to 3D reconstruction using ab initio reconstruction and non-uniform refinement without any symmetry imposition to validate whether the particles were sorted correctly. Among those, the one with four bound Gα_i3_ proteins was further refined using non-uniform refinement with C4 symmetry at a GSFSC resolution of 3.54 Å using CryoSPARC. In each map processed with either C1 or C4, the density of the binding interface and Gα_i3_ was still weak. Several rounds of focused classifications of Gα_i3_-bound subtracted particles were performed in Relion, and the particles with the best class were transferred back to CryoSPARC for local refinement, which yielded a GSFSC resolution of 4.19 Å. A composite map of the fully occupied TRPC5-Gα_i3_ complex (TRPC5_Class1_-Gα_i3_) was made from the TRPC5-Gα_i3_ map with four Gα_i3_ proteins processed with C4 symmetry and the four copies of focused ARD-Gα_i3_ maps using the ‘vop maximum’ command in UCSF Chimera^66^. Further 3D classification of the particles of the fully occupied TRPC5-Gα_i3_ complex yielded another class for the TRPC5-Gα_i3_ conformation, which was refined at a GSFSC resolution of 3.79 Å and also used to make a composite map of TRPC5_Class2_-Gα_i3_ complex. Local resolutions for all maps were assessed by the local resolution estimation in CryoSPARC.

For TRPC5 in lipid nanodiscs, 5,523 collected movies were subjected to motion correction and CTF estimation using patch motion correction and patch CTF in CryoSPARC. After several rounds of 2D classifications, 563,703 particle images were selected and subjected to 3D reconstruction using ab initio reconstruction and non-uniform refinement with C4 symmetry imposed, resulting in a consensus map of 3.27 Å GSFSC resolution. To improve the map quality of the cytosolic domain, heterogeneous refinement was performed, and the best class was selected and re-extracted with a box size of 400 pixels. The re-extracted particles were further refined by non-uniform refinement, which yielded a map with a final 3.15 Å GSFSC resolution (TRPC5_Class1_). Moreover, cytosolic domain-focused 3D classification of the particles used for the reconstruction of the consensus map in Relion yielded other classes for TRPC5 with about 30% of distribution (TRPC5_Class2_). The particles were further 3D-classified, and particles for the best class were re-extracted with a box size of 400 pixels and refined in CryoSPARC, resulting in a GSFSC resolution of 3.59 Å (TRPC5_Class2_). Local resolution for the map was assessed by the local resolution estimation in CryoSPARC.

### Model building, refinement, and validation

For the atomic models of the TRPC5_Class1_-Gα_i3_ and TRPC5_Class2_-Gα_i3_ complexes, PDB entries 7E4T (human TRPC5 apo state structure at 3 angstrom)^25^ and 2ODE (crystal structure of the heterodimeric complex of human RGS8 and activated Gi alpha 3)^67^ were used to generate initial templates for model building. The models of TRPC5 and Gα_i3_ were fitted into the composite EM density maps using UCSF chimera. The overall TRPC5, the binding interface, and the GTP-binding region of the Gα_i3_ was manually adjusted in Coot^68^ while the rest of the Gα_i3_ remained mostly unmodified. The ligands and lipids were manually adjusted in Coot. The overall model was further refined and validated by Phenix^69^.

For the atomic models of the TRPC5_Class1_ and TRPC5_Class2_, a similar strategy mentioned above was applied; PDB entries 7E4T (human TRPC5 apo state structure at 3 angstrom)^25^ was used to generate initial templates for model building. After fitting the model to the EM densities, the ligands, lipids, and the models were manually adjusted in Coot and further refined and validated by Phenix.

All structural figures and movies were visualized and generated by PyMOL, UCSF Chimera^66^, or UCSF ChimeraX^70^.

### Molecular dynamics (MD) simulation and analysis

For initial model generation, missing loops of TRPC5 (7X6C) and TRPC5-Gα_i3_ (7X6I) were filled in Coot using AlphaFold-predicted structures as references. For initial models with PIP_2_, diC8-PIP_2_ was manually docked into the putative PIP_2_ binding site of the TRPC5 model (7X6C, this study) in Coot, and energy-minimized using AutoDock Vina software^71,72^. Each model showing the best score was chosen for the initial model for further MD simulations.

Three MD simulation systems, including TRPC5, TRPC5-PIP_2_, and TRPC5-Gα_i3_ were built by embedding the TRPC5 or TRPC5-Gα_i3_ complex into a mixed membrane of POPC: POPE: Cholesterol = 2:1:1. Sodium and chloride ions were added to neutralize the charge of the entire simulation system and maintain a salt concentration of ~0.15 M. The PIP_2_ ligands were added to the proposed binding sites in TRPC5-PIP_2_ system, and were modeled as SAPI24, phosphatidylinositol-4,5-bisphosphate with protonation on 4′-phosphate group and stearic (18:0) and arachidonic (20:4) acid as tails. The simulations were performed using the CHARMM36m force field (lipid and protein)^73–76^, TIP3P water model^77^. The initial simulation system was assembled in CHARMM-GUI Membrane Builder^78–81^ and equilibrated using the standard Membrane Builder six-step protocol. After that, additional restrained simulation of 30 ns was performed for protein backbone to relax the whole protein, with the harmonic force constant gradually reducing from 50 to 0 kJ mol^−1^ nm^−2^. Further unrestrained production of 70 ns was performed in OpenMM^82^ until shifting to Anton2^83^ for extended simulations of 500 ns. Therefore, a total of 600 ns simulation trajectory is available for each simulation system. The simulations were performed under the NPT (constant particle number, 1 bar pressure, and 300 K temperature) condition, with Multigrator integrator for pressure coupling and Nosé-Hoover method for temperature coupling^84,85^. A time step of 2 fs and a frame saving frequency of every 240 ps were used for Anton2 simulations. The van der Waals interactions were cut off at 12 Å with a force-switching function between 10 and 12 Å.

For PIP_2_ RMSD analysis, the heavy atoms of polar head group and glycerol region were selected to derived ligand RMSD. The PIP_2_-binding residue distance is defined as the distance between the center-of-mass of PIP_2_ inositol ring and the center of mass of the binding residue side chain. The TRPC5-Gα_i3_ binding stability is characterized by IYY-Gα_i3_ distance, which is based on backbone of residues 57–59 (IYY) in TRPC5 and residues R208/I212 in Gα_i3_. Visualization movies of simulation trajectory were made with VMD^86^.

### Reporting summary

Further information on research design is available in the Nature Portfolio Reporting Summary linked to this article.

## Supplementary information

Supplementary Information

Peer Review File

Description of Additional Supplementary Files

Supplementary Movie 1

Supplementary Movie 2

Supplementary Movie 3

Supplementary Movie 4

Reporting Summary

## Data Availability

The cryo-EM density maps and corresponding atomic coordinates for the TRPC5_Class1_, TRPC5_Class1_-Gα_i3_, TRPC5_Class2_ and TRPC5_Class2_-Gα_i3_ have been deposited in the Electron Microscopy Data Bank (EMDB) and Protein Data Bank (PDB) under EMDB accession codes EMD-33021, EMD-33022, EMD-34300, EMD-34301 and under PDB accession codes 7X6C, 7X6I, 8GVW, 8GVX, respectively. PDB entries 7E4T (human TRPC5 apo state structure at 3 Å) and 2ODE (crystal structure of the heterodimeric complex of human RGS8 and activated Gi alpha 3) were used to generate the initial template for model building. The MD simulation data generated in this study have been deposited in the Zenodo OpenAIRE database under accession code 7768238. The source data underlying Figs. 2b, h, i, 4e, f, j, 5i–k, Supplementary Figs. 1e, 5d, g, 9d–f and h are provided as a Source Data file. Uncropped scans of all gels and blots in the Supplementary Figures are provided at the end of the Supplementary Information. Source data are provided in this paper.

## Source data

Source Data
