# Supplementary material for: Molecular architecture of the Gαi-bound TRPC5 ion channel
Source: Nat Commun. 2023 May 3;14:2550. doi: 10.1038/s41467-023-38281-3 (PMC10156788; doi:10.1038/s41467-023-38281-3)
Supplement: Supplementary file 1 — Supplementary Information [file 41467_2023_38281_MOESM1_ESM.pdf]

Supplementary Information

Molecular architecture of the Gα_i-bound TRPC5 ion channel

Jongdae Won, Jinsung Kim et al.

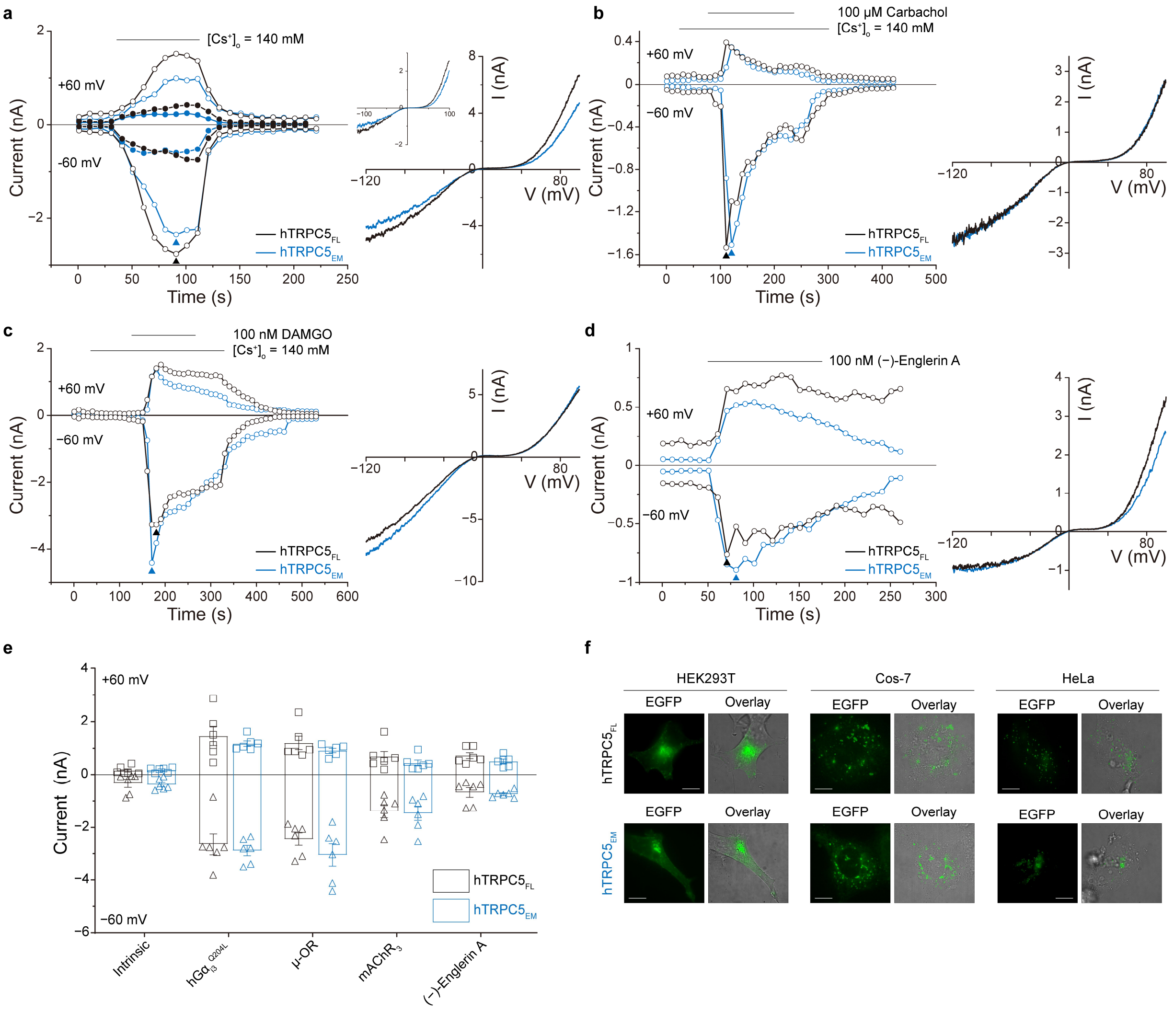

Supplementary Fig. 1. Functional validation of the full-length human TRPC5 (TRPC5$_{FL}$) channel and truncated human TRPC5 (TRPC5$_{EM}$) channel used for cryo-EM. The electrophysiological characteristics of the two channel constructs were examined through physiological activators (G_q- or G_i-signaling) or a pharmacological agonist [(−)-Englerin A] in HEK293T cells. The current-voltage (I-V) curve was from a ramp pulse at a specific time point annotated as triangles. **a**, Intrinsic activities (*filled circles*) of TRPC5 channels were measured using Cs-rich extracellular solution ($[Cs^+]_o$ = 140 mM, for complete compositions of extracellular

solutions, see *Methods*). The coexpression of the GTPase-deficient form of the alpha subunit of human inhibitory G-protein subtype 3 ($hG\alpha_{i3}^{Q204L}$) elicited a much larger cationic current (*open circles*) in both full-length and truncated channels. Inset I-V curves were from intrinsic currents. **b**, Coexpressed with human muscarinic acetylcholine receptor subtype 3 ($mAChR_3$), both full-length and truncated channels showed doubly-rectifying cationic current by extracellular carbachol (100 μM), a potent ACh analog. **c**, Similar to (**b**), robust TRPC5 currents were measurable in cells coexpressing human mu-opioid receptor (μ-OR) when 100 nM of DAMGO, a mu-opioid receptor agonist, was applied extracellularly. **d**, 100 nM (−)-Englerin A, a specific and potent TRPC4 or 5 channel activator, induced a strong cationic current for both full-length and truncated channels. **e**, A summarized current amplitude at ± 60 mV. No significant difference in current amplitudes was observable between full-length and truncated channels regardless of activation systems. The *p* values for differences between two channels at each condition are 0.791, intrinsic; 0.458, $hG\alpha_{i3}^{Q204L}$; 0.313, μ-OR; 0.314, $mAChR_3$; and 0.169, (−)-Englerin A for currents at +60 mV and 0.786, intrinsic; 0.596, $hG\alpha_{i3}^{Q204L}$; 0.239, μ-OR; 0.830, $mAChR_3$, and 0.748, (−)-Englerin A for currents at −60 mV. Bars represent the means ± s.e.m. ($n = 6$ for each condition). *p* values were calculated in two-sided Student's t-test. **f**, Epi fluorescence and DIC overlay images of both channels in transiently transfected HEK293T, Cos-7 and HeLa cells (scale bars, 10 μm). The expression patterns, i.e., the intracellular localizations of the two channel proteins, were similar in all three cell types. Six independent images were acquired in all three cell types.

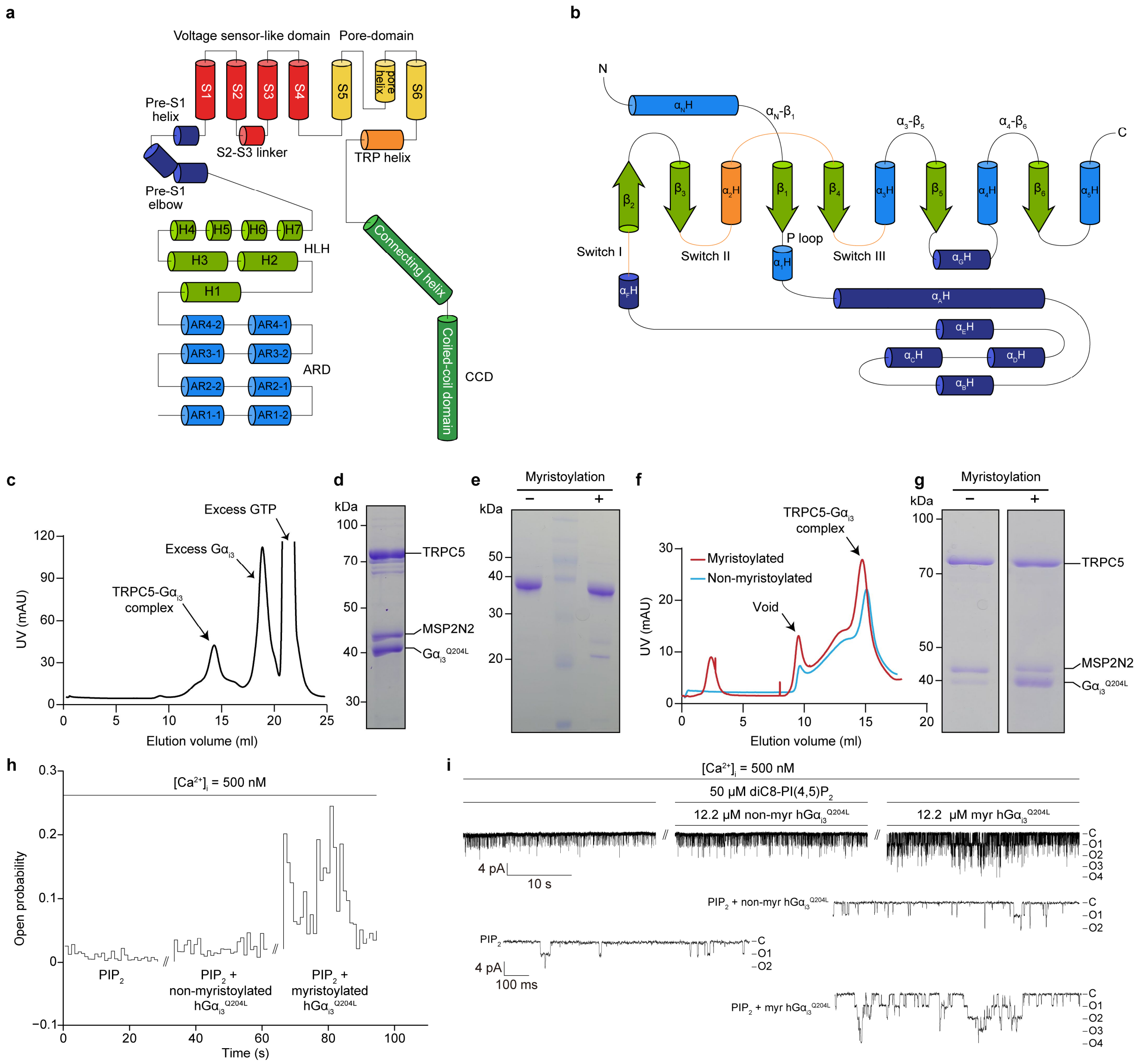

Supplementary Fig. 2. Domain architectures of the TRPC5 and Gαi3, purification of the TRPC5-Gαi3 complex, and biochemical and electrophysiological evidence for the effect of myristoylation on the TRPC5-Gαi3 complex. a, Domain architecture of the human TRPC5 channel. ARD, ankyrin repeat domain; HLH, helix-loop-helix; CCD, coiled-coil domain. **b**, Domain architecture of the human Gαi3. **c**, **d**, Purification of the TRPC5-Gαi3 complex; representative size-exclusion chromatogram (**c**) and SDS–PAGE (**d**). More than ten independent

experiments of the complex purification were carried out with consistency. **e**, Validation of myristoylation in Gα_{i3}. Myristoylated Gα_{i3} shows faster electrophoretic mobility than non-myristoylated Gα_{i3}. SDS–PAGE was performed on a gel supplemented with 4 M urea. More than ten independent experiments of the purification of Gα_{i3} (myristoylated and non-myristoylated) were carried out with consistency. **f**, **g**, Effect of myristoylation on TRPC5-Gα_{i3} binding; size-exclusion chromatograms (**f**) and SDS–PAGE of the peak fraction (**g**). Nanodisc-reconstituted TRPC5 was divided into two groups, each was mixed with myristoylated or non-myristoylated Gα_{i3} and subjected to size-exclusion chromatography. Myristoylated Gα_{i3} was coeluted with TRPC5 at an earlier retention time in size-exclusion chromatography (**f**) and showed stronger intensity in SDS–PAGE (**g**) indicating that the binding event occurs more favorably when the Gα_{i3} was myristoylated. Three independent experiments of the analytical size-exclusion chromatography and SDS–PAGE analysis were performed, and the results were consistently reproduced. **h**, Open probability trace with respect to each intracellular condition. **i**, Representative current trace from the excised patch according to intracellular conditions. Below are current traces with an expanded time scale. Non-myr, non-myristoylated; myr, myristoylated.

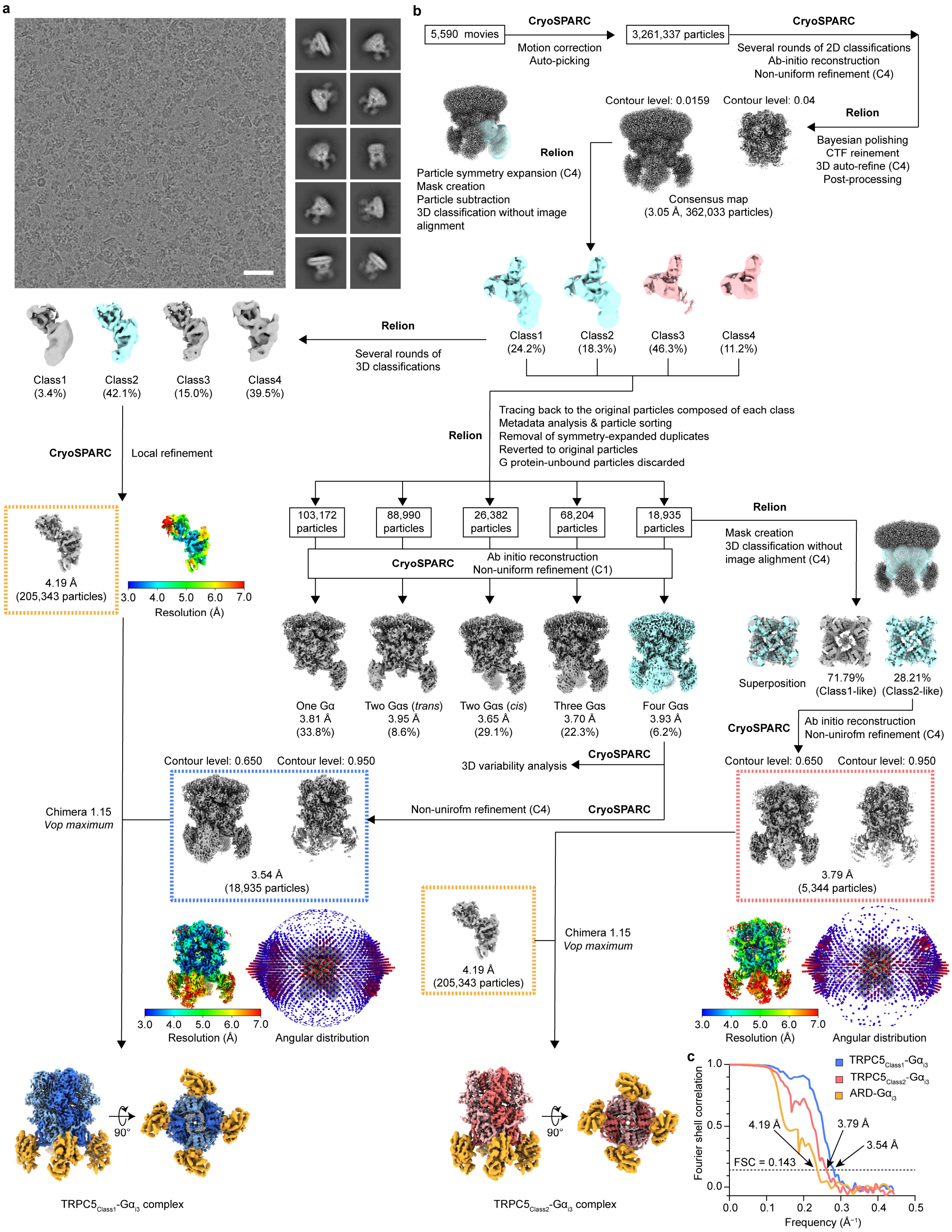
a
b
5,590 movies
CryoSPARC
Motion correction
Auto-picking
3,261,337 particles
CryoSPARC
Several rounds of 2D classifications
Ab-initio reconstruction
Non-uniform refinement (C4)
Contour level: 0.0159
Contour level: 0.04
Relion
Bayesian polishing
CTF reinement
3D auto-refine (C4)
Post-processing
Consensus map
(3.05 Å, 362,033 particles)
Relion
Particle symmetry expansion (C4)
Mask creation
Particle subtraction
3D classification without image alignment
Class1 (24.2%)
Class2 (18.3%)
Class3 (46.3%)
Class4 (11.2%)
Relion
Several rounds of 3D classifications
Class1 (3.4%)
Class2 (42.1%)
Class3 (15.0%)
Class4 (39.5%)
CryoSPARC
Local refinement
Relion
Tracing back to the original particles composed of each class
Metadata analysis & particle sorting
Removal of symmetry-expanded duplicates
Reverted to original particles
G protein-unbound particles discarded
103,172 particles
88,990 particles
26,382 particles
68,204 particles
18,935 particles
CryoSPARC
Ab initio reconstruction
Non-uniform refinement (C1)
Relion
Mask creation
3D classification without image alighment (C4)
4.19 Å
(205,343 particles)
3.0 4.0 5.0 6.0 7.0
Resolution (Å)
One Gα 3.81 Å (33.8%)
Two Gαs (trans) 3.95 Å (8.6%)
Two Gαs (cis) 3.65 Å (29.1%)
Three Gαs 3.70 Å (22.3%)
Four Gαs 3.93 Å (6.2%)
Superposition
71.79% (Class1-like)
28.21% (Class2-like)
CryoSPARC
Ab initio reconstruction
Non-unirofm refinement (C4)
CryoSPARC
3D variability analysis
Contour level: 0.650
Contour level: 0.950
Chimera 1.15
Vop maximum
Non-unirofm refinement (C4)
CryoSPARC
3.54 Å
(18,935 particles)
Contour level: 0.650
Contour level: 0.950
3.79 Å
(5,344 particles)
4.19 Å
(205,343 particles)
Chimera 1.15
Vop maximum
3.0 4.0 5.0 6.0 7.0
Resolution (Å)
Angular distribution
3.0 4.0 5.0 6.0 7.0
Resolution (Å)
Angular distribution
c
TRPC5Class1-Gαi3
TRPC5Class2-Gαi3
ARD-Gαi3
Fourier shell correlation
1.0
0.5
0.0
4.19 Å
3.79 Å
3.54 Å
FSC = 0.143
0.0 0.1 0.2 0.3 0.4 0.5
Frequency (Å⁻¹)
90°
TRPC5Class1-Gαi3 complex
90°
TRPC5Class2-Gαi3 complex

Supplementary Fig. 3. Cryo-EM data processing of the TRPC5-Gα_{i3} complexes. a, Representative cryo-EM micrograph from 5,590 movies (scale bar, 50 nm) and 2D class averages of the TRPC5-Gα_{i3} complexes. **b**, Workflow of cryo-EM data processing of the TRPC5-Gα_{i3} complexes. All indicated contour levels are based on visualization using UCSF Chimera[1]. **c**, Gold-standard Fourier shell correlation curves of the inputs for the composite map: TRPC5$_{Class1}$-Gα_{i3} (blue), TRPC5$_{Class2}$-Gα_{i3} (red) and locally refined Gα_{i3} (yellow).

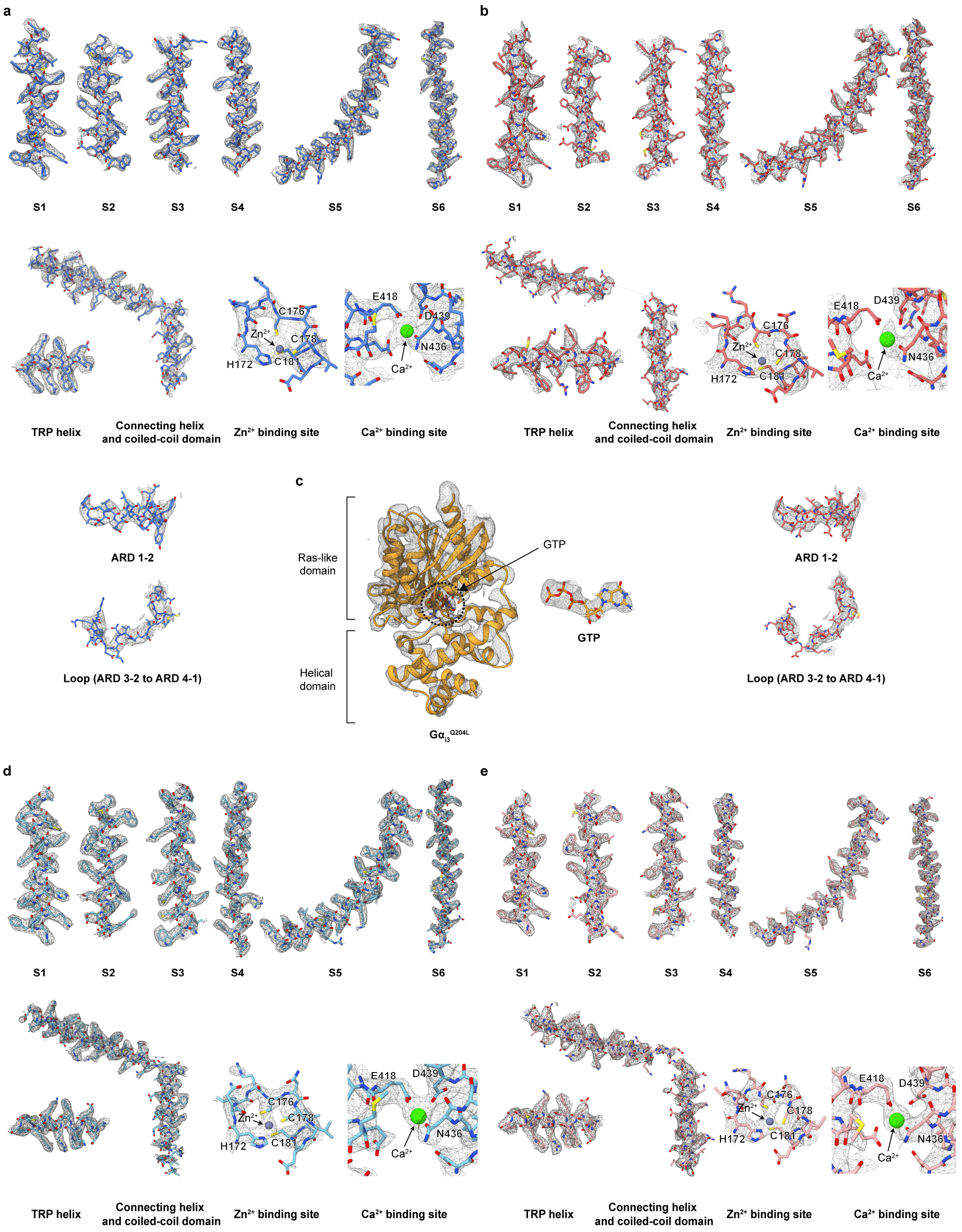

a
S1
S2
S3
S4
S5
S6
E418
D439
C176
Zn2+
C178
N436
H172
C181
Ca2+
TRP helix
Connecting helix and coiled-coil domain
Zn2+ binding site
Ca2+ binding site
b
S1
S2
S3
S4
S5
S6
E418
D439
C176
Zn2+
C178
N436
H172
C181
Ca2+
TRP helix
Connecting helix and coiled-coil domain
Zn2+ binding site
Ca2+ binding site
ARD 1-2
Loop (ARD 3-2 to ARD 4-1)
c
Ras-like domain
Helical domain
GTP
GTP
Gαi3 Q204L
ARD 1-2
Loop (ARD 3-2 to ARD 4-1)
d
S1
S2
S3
S4
S5
S6
E418
D439
C176
Zn2+
C178
N436
H172
C181
Ca2+
TRP helix
Connecting helix and coiled-coil domain
Zn2+ binding site
Ca2+ binding site
e
S1
S2
S3
S4
S5
S6
E418
D439
C176
Zn2+
C178
N436
H172
C181
Ca2+
TRP helix
Connecting helix and coiled-coil domain
Zn2+ binding site
Ca2+ binding site

Supplementary Fig. 4. Density map features in the cryo-EM structures of TRPC5-Gα_{i3} complexes and TRPC5 in lipid nanodiscs. a–d, Representative EM densities for various parts in TRPC5$_{Class1}$-Gα_{i3} (**a**), TRPC5$_{Class2}$-Gα_{i3} (**b**), Gα_{i3} in the TRPC5-Gα_{i3} complexes (**c**), TRPC5$_{Class1}$ (**d**) and TRPC5$_{Class2}$ (**e**).

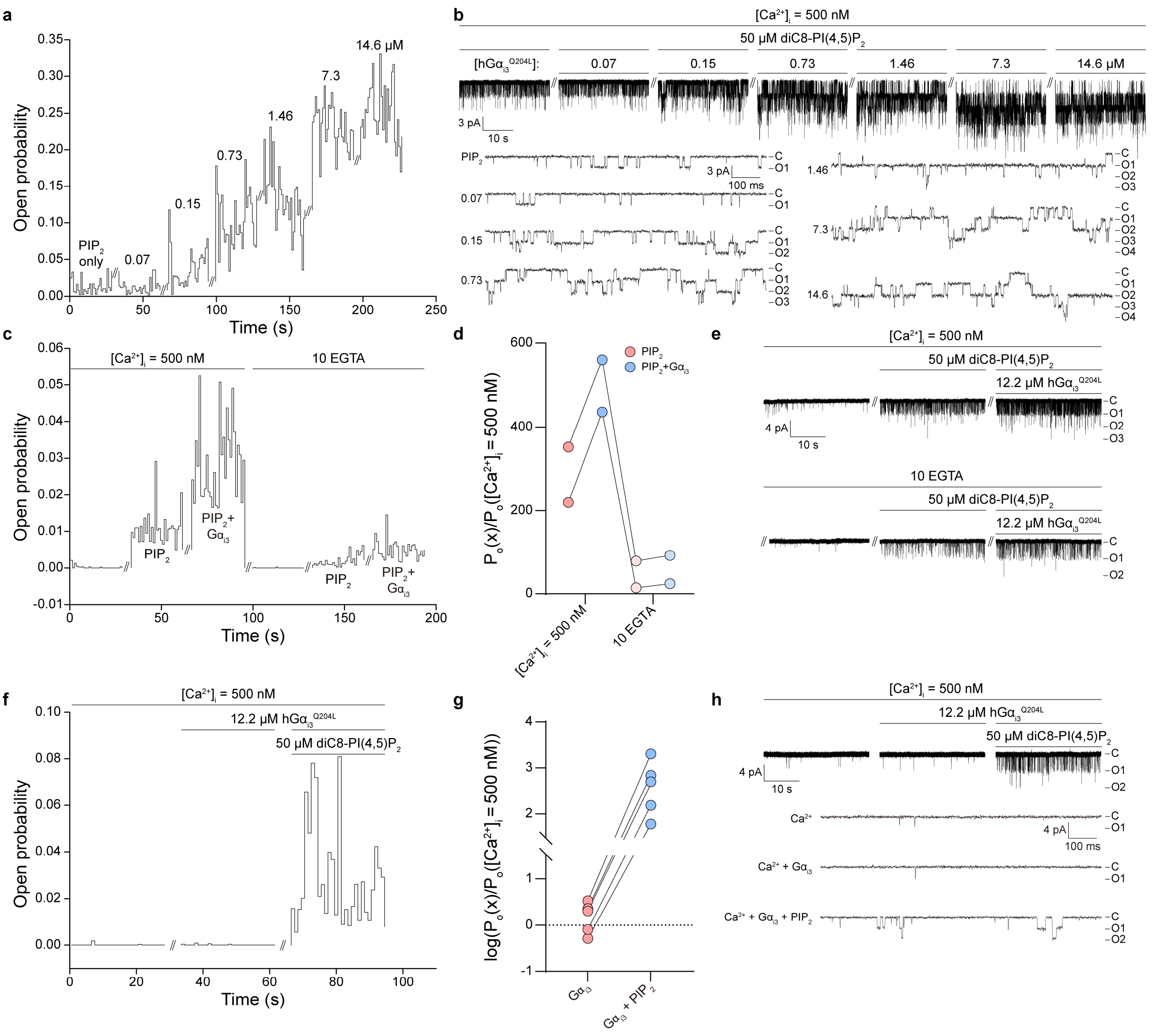

Supplementary Fig. 5. Functional interplay among Ca^{2+}, PIP_2, and $G\alpha_{i3}$ in the activation process of the TRPC5 channel. a, Open probability trace with respect to the concentration of purified $G\alpha_{i3}^{Q204L}$ proteins. Fifty micromolar PIP_2 was included in all protein samples to prevent run-down. **b**, Representative current trace from the excised patch. Below are current traces at each intracellular condition with an expanded time scale. **c**, Open probability trace according to each intracellular condition. The 10 EGTA solution contained 10 mM EGTA but no additional calcium chloride; therefore, only a trace amount of chelator-free calcium was expected to be present in the solution. **d**, Fold-increase in open probability with respect to the mean open probability at $[Ca^{2+}]_i$

= 500 nM. **e**, A representative current trace is shown. **f**, Change in open probability with respect to each intracellular condition. **g**, **h**, Fold-increase in open probability (**g**) and representative current trace (**h**) are shown as in (**d**) and (**e**).

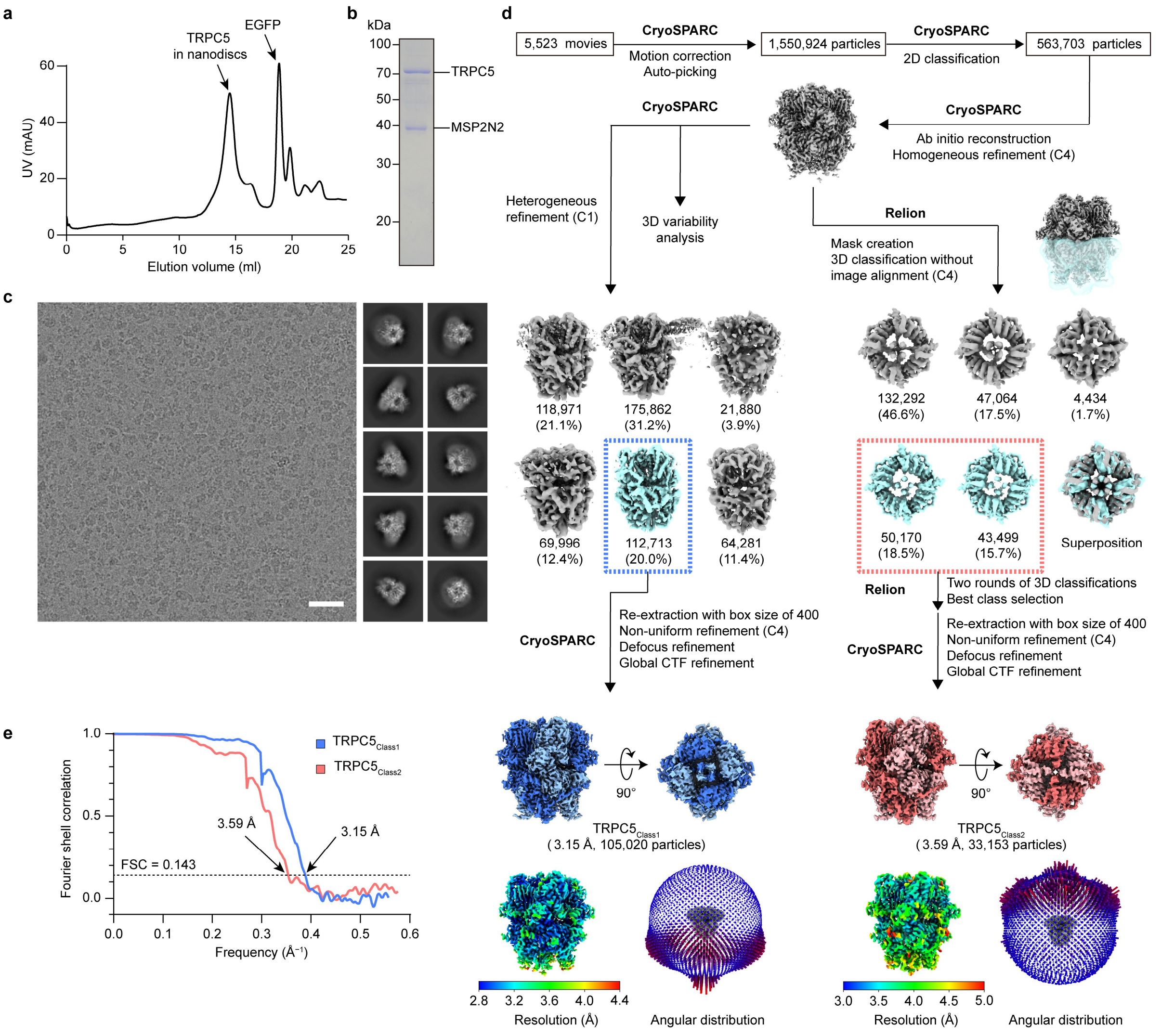

Supplementary Fig. 6. Purification and cryo-EM data processing of TRPC5 structures in lipid nanodiscs. a, **b**, Purification of TRPC5 in lipid nanodiscs; representative size-exclusion chromatogram (**a**) and SDS–PAGE (**b**). Four independent experiments of the purification of the TRPC5 in lipid nanodiscs were carried out with consistency. **c**, Representative cryo-EM micrograph from 5,523 movies (scale bar, 50 nm) and 2D class averages of TRPC5 in lipid nanodiscs. **d**, Workflow of cryo-EM data processing of $TRPC5_{Class1}$ and $TRPC5_{Class2}$ in lipid

nanodiscs. **e**, Gold-standard Fourier shell correlation curve for the 3D reconstructions of TRPC5 in lipid nanodiscs.

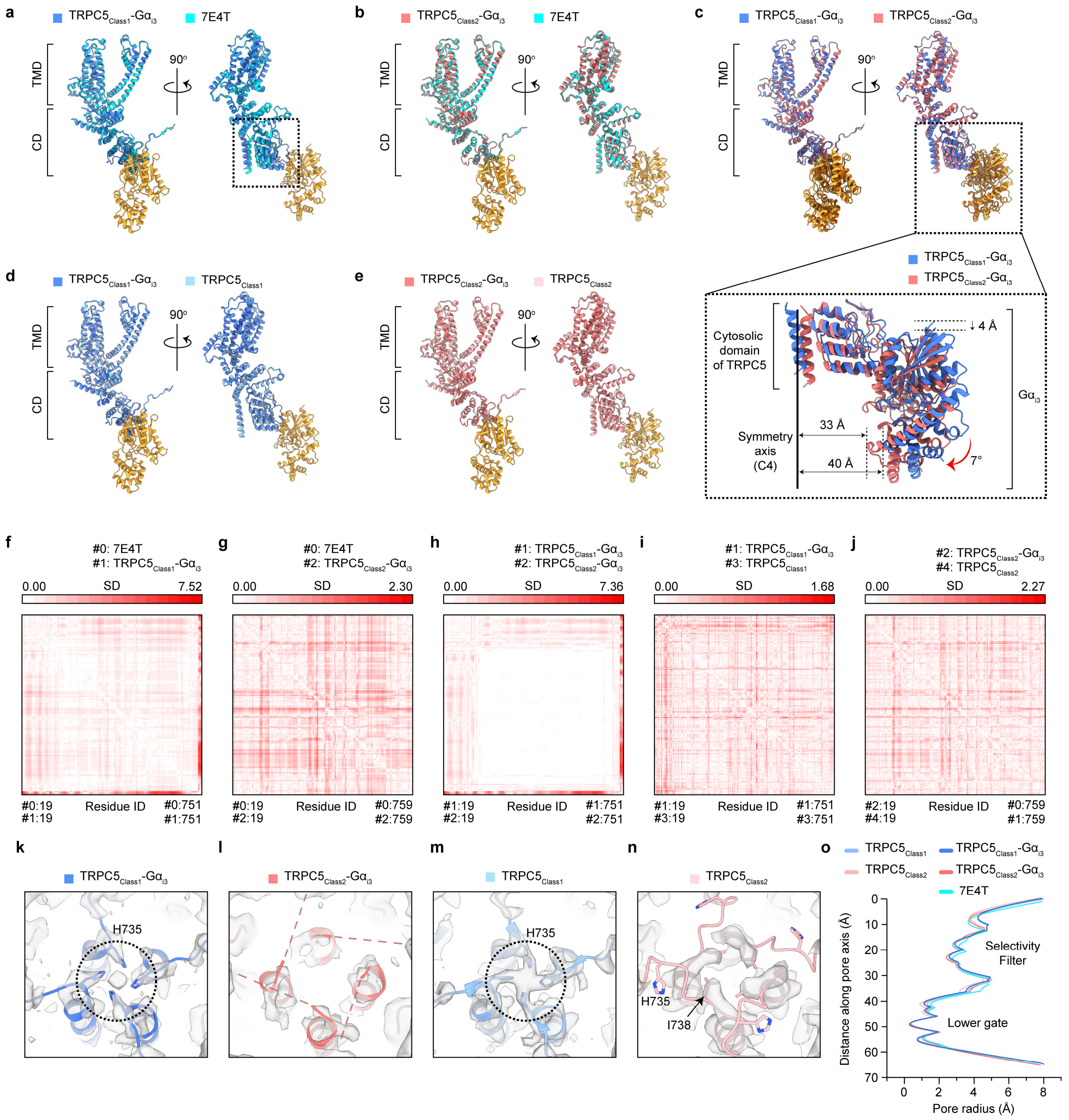

Supplementary Fig. 7. Structural comparisons of the TRPC5 and TRPC5-Gα$_{i3}$ complexes. **a–e**, Structural comparisons of single chains from TRPC5$_{Class1}$-Gα$_{i3}$ and TRPC5 in detergent (7E4T) (**a**), TRPC5$_{Class2}$-Gα$_{i3}$ and TRPC5 in detergent (7E4T) (**b**), TRPC5$_{Class1}$-Gα$_{i3}$ and TRPC5$_{Class2}$-Gα$_{i3}$ (**c**), TRPC5$_{Class1}$-Gα$_{i3}$ and TRPC5$_{Class1}$ (**d**), and TRPC5$_{Class2}$-Gα$_{i3}$ and

TRPC5$_{Class2}$ (**e**). The RMSD values over Cα atoms in one subunit between two structures are 1.841 Å (**a**), 0.557 Å (**b**), 1.728 Å (**c**), 0.456 Å (**d**), and 0.514 Å (**e**), respectively. Structural differences are highlighted in the dotted box (**a**, **c**). A close-up view and difference details of Gα_{i3} are shown in the dotted box in (**c**) at the bottom. **f**–**j**, Difference distance matrix plots with two structures. 2D labels for standard deviation are depicted above the plots. The comparison sets are as follows: TRPC5-detergent with TRPC5$_{Class1}$-Gα_{i3} (**f**), TRPC5-detergent with TRPC5$_{Class2}$-Gα_{i3} (**g**), TRPC5$_{Class1}$-Gα_{i3} with TRPC5$_{Class2}$-Gα_{i3} (**h**), TRPC5$_{Class1}$-Gα_{i3} with TRPC5$_{Class1}$ (**i**), and TRPC5$_{Class2}$-Gα_{i3} with TRPC5$_{Class2}$ (**j**). **k**–**n**, Close-up views of the connecting helix-coiled-coil junction in TRPC5$_{Class1}$-Gα_{i3} (**k**), TRPC5$_{Class2}$-Gα_{i3} (**l**), TRPC5$_{Class1}$ (**m**), TRPC5$_{Class2}$ (**n**). Distinct metal-like densities coordinated by four H735 residues are highlighted in dotted circles. Dotted lines in (**l**) indicate unmodelled region between connecting helix and coiled-coil domain. **o**, Pore radii along the central axis in the TRPC5 and TRPC5-Gα_{i3} complexes calculated from the HOLE program[2]. All structures show closed conformations at the lower gate. TMD, transmembrane domain; CD, cytosolic domain.

a

Purpose	Template	Mutation	I (nA)[1]	*n*	*p*-value	Comment
ARD-CCD interaction	$hTRPC5_{EM}$	wild-type	−0.38 ± 0.09	6	control	
		N80A	−1.77 ± 0.23	4	0.0057	
		N82A	−1.48 ± 0.11	3	0.0010	
		N80A/N82A	N/A	N/A	N/A	not expressed[2]
		H735A	−0.61 ± 0.29	4	0.4982	
		E736A	−0.99 ± 0.48	5	0.2780	
		H735A/E736A	−0.56 ± 0.12	4	0.2951	
		Q748A	−0.10 ± 0.05	6	0.0198	
		Q748R	−1.35 ± 0.34	5	0.0449	
		N80A/N82A/Q748A	N/A	N/A	N/A	not expressed[2]
TRPC5-Gα_{i3} binding interface | close contact	$hTRPC5_{EM}$	wild-type[3]	−2.89 ± 0.19	6	control	co-expressed with hGα_{i3}^{Q204L}
		I57A	−2.05 ± 0.76	6	0.3288	
		Y58A	−2.01 ± 0.61	6	0.2194	
		Y59A	−3.44 ± 0.67	6	0.4604	
		I57A/Y58A	−0.86 ± 0.36	6	0.0013	
		I57A/Y59A	−1.59 ± 0.22	6	0.0005	
		Y58A/Y59A	−0.85 ± 0.32	6	0.0014	
	hGα_{i3}^{Q204L}	wild-type	−2.56 ± 0.31	6	control	co-expressed with $hTRPC5_{EM}$
		R205A	−1.99 ± 0.18	6	0.1501	
		R208A	−0.54 ± 0.23	5	0.0006	
		R205A/R208A	−0.67 ± 0.12	8	0.0009	
		E245A	−1.30 ± 0.38	6	0.0295	
		K248A	−1.49 ± 0.27	6	0.0276	
		R205A/E245A	−1.55 ± 0.34	8	0.0501	
		E245A/K248A	−1.02 ± 0.14	10	0.0026	
		R205A/E245A/K248A	−0.13 ± 0.03	6	0.0005	
		W211A	−1.51 ± 0.18	6	0.0193	
		I212A	−2.10 ± 0.90	7	0.2039	
		F215A	−2.06 ± 0.19	6	0.2045	
		W211A/I212A	−2.76 ± 0.68	6	0.7945	
		I212A/F215A	−2.52 ± 0.63	6	0.9590	
		W211A/F215A	−1.45 ± 0.26	5	0.0230	
		W211A/I212A/F215A	−1.70 ± 0.32	9	0.0763	
		R205A/R208A/W211A/I212A/F215A	−0.60 ± 0.17	10	0.0005	

[1] Currents measured at −60 mV. mean ± s.e.m.
[2] Neither fluorescence signal in plasma membrane nor distinguishable whole-cell current was observable.
[3] Numbers from Fig.4

b

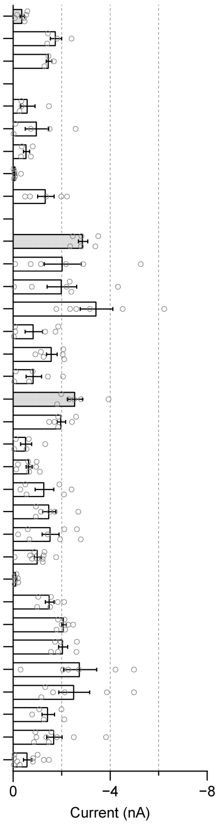

Supplementary Fig. 8. List of mutations assessed for the ARD-CCD interaction within TRPC5 channels, and the binding interface between TRPC5 and the Gα_{i3} protein. a, Tables show the purpose of the mutation, template and mutation sites, the mean ± s.e.m. of current amplitudes, number of recordings, and p values from one-sided Student's t test with respect to the wild-type group for each purpose. **b**, Summary of current amplitudes is shown in bar graphs. Circles represent current amplitudes from independent recordings. The wild-type groups at each purpose are shaded in gray color.

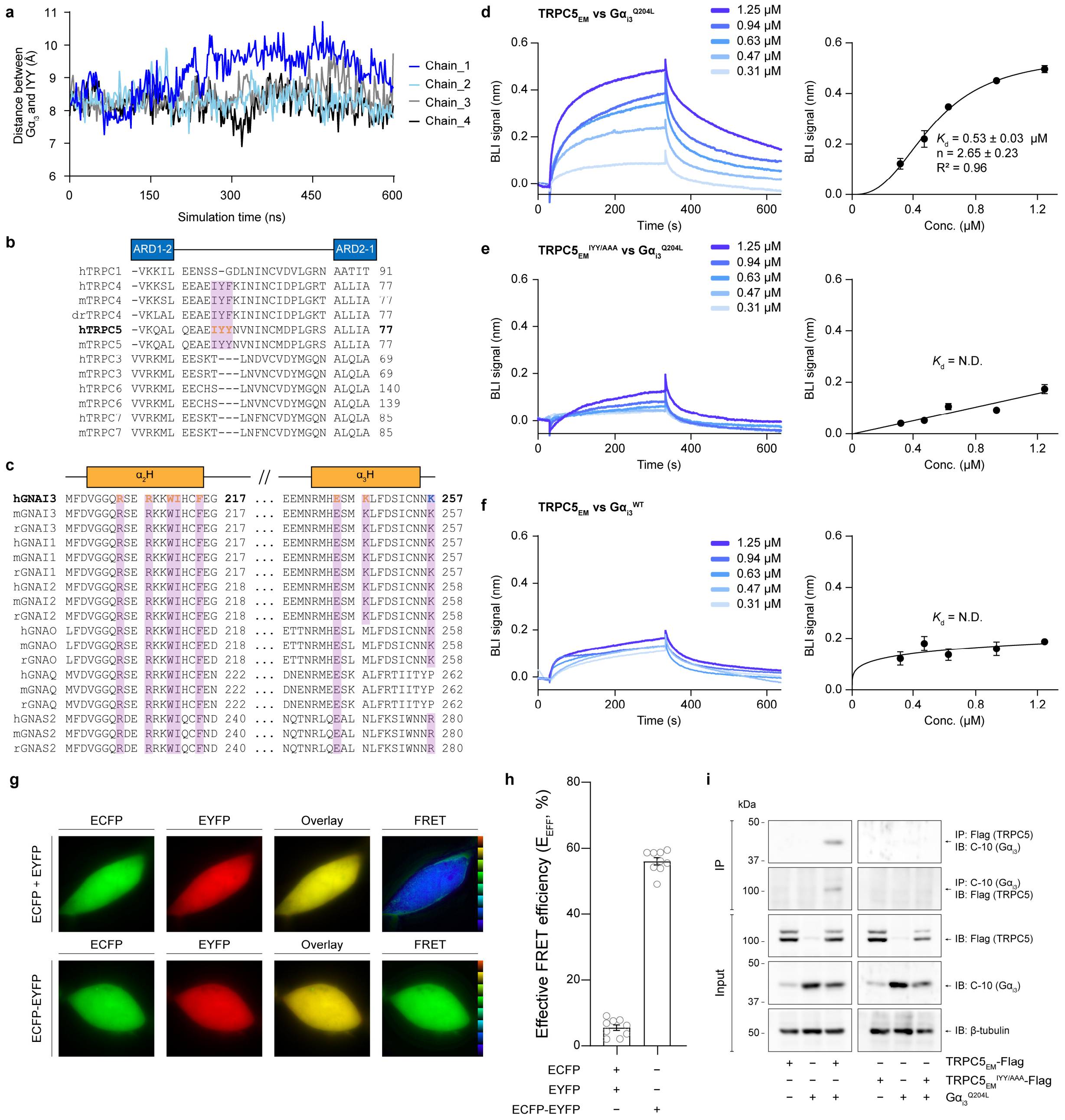

Supplementary Fig. 9. Contribution of the IYY motif to complex formation between TRPC5 and Gαi3. a, The distance between the IYY motif in TRPC5 and Gαi3 plotted as a function of simulation time in MD simulation. **b**, **c**, Sequence alignment of TRPC (**b**) and Gα (**c**). The preceding lowercase for each sequence name indicates species (h, human; m, mouse; r, rat; dr,

Danio renio also known as zebrafish). For the sequence name of Gα proteins, a genetic nomenclature was adopted (GNAI, G alpha i; GNAO, G alpha o; GNAQ, G alpha q; GNAS2, G alpha s). The purple shading highlights the conserved sequence compared to the bolded sequence (human TRPC5 or human $G\alpha_{i3}$). For both TRPC and Gα proteins, letters highlighted with orange color are residues that may be important for establishing close contact interactions through the IYY motif. A letter highlighted with blue color is a candidate residue for mediating long-range electrostatic interactions. **d–f**, Biolayer interferometry (BLI) assays of $TRPC5_{EM}$ vs. N-myristoylated $G\alpha_{i3}^{Q204L}$ (**d**), $TRPC5_{EM}^{IYY/AAA}$ vs. N-myristoylated $G\alpha_{i3}^{Q204L}$ (**e**), and $TRPC5_{EM}$ vs. N-myristoylated $G\alpha_{i3}^{WT}$ (**f**). BLI sensorgrams with each concentration of $G\alpha_{i3}$ are shown in different colors (left). Data were fitted to the Hill's equation (right). Symbols and bars represent the mean ± s.e.m. of n = three independent experiments. **g**, Epi fluorescence images, overlay images, and FRET images from each expression pair. Images from cells coexpressing ECFP and EYFP (ECFP + EYFP) or cells expressing artificially linked ECFP-EYFP fusion protein (ECFP-EYFP) are shown as a qualitative calibration for FRET imaging. Colored E_{EFF} indicators on the right side of FRET images were set to cover from 0% (dark blue) to 100% (red) linearly. Ex, excitation; Em, emission. **h**, Summary of E_{EFF} from each expression pair (n = 9, ECFP + EYFP; n = 9, ECFP-EYFP). Bars represent the mean ± s.e.m. **i**, $TRPC5_{EM}$-Flag, $TRPC5_{EM}^{IYY/AAA}$-Flag, and $G\alpha_{i3}^{Q204L}$ were either expressed alone or coexpressed in HEK293T cells, as shown below the blots. Five hundred micrograms of proteins from each condition was subjected to immunoprecipitation with anti-Flag antibody and probed with an antibody against $G\alpha_{i3}$ proteins (C-10), or vice versa.

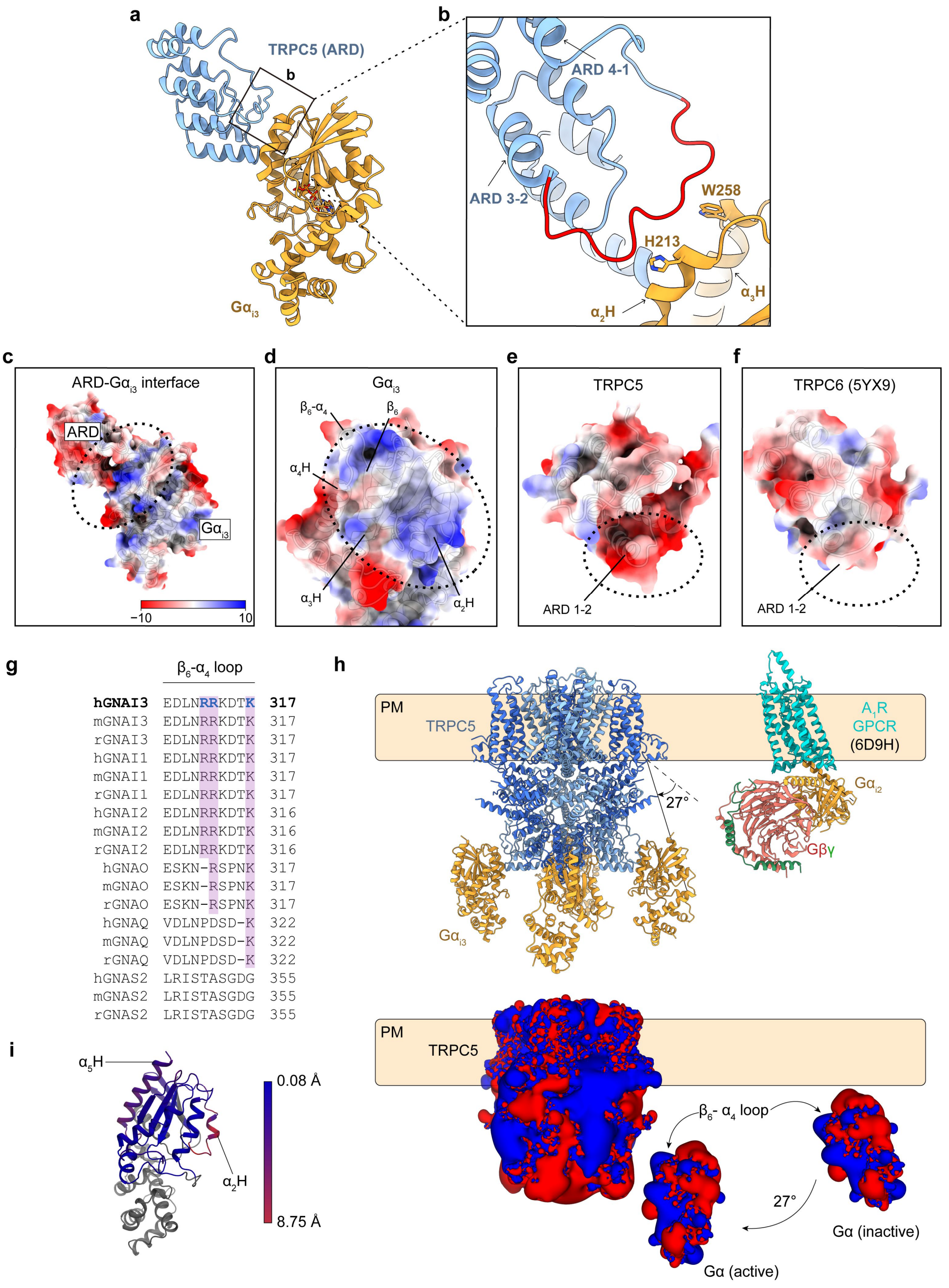

Supplementary Fig. 10. Possible involvement of loop interactions and long-range electrostatic interactions in TRPC5-Gα$_{i3}$ binding. a, Side view of an atomic model focused on the ARD of TRPC5 and Gα$_{i3}$. The box indicates the TRPC5-Gα$_{i3}$ interface expanded in (**b**). **b**, Close-up view of the interface between the loop region of TRPC5 and Gα$_{i3}$ with putative

interacting residues on $G\alpha_{i3}$ (H213 and W258). **c–f**, Electrostatic (Coulombic) surfaces (scale in kcal mol^{-1} e^{-1}) of ARD-$G\alpha_{i3}$ (**c**), $G\alpha_{i3}$ (**d**), ARD of TRPC5 (**e**) and TRPC6 (**f**)[3]. Complementary electrostatic interaction (**c**) and electropositive (**d**), electronegative (**e**) and electroneutral (**f**) charges are highlighted in dotted circles. **g**, Sequence alignment of β_6-α_4 loop of Gα. The preceding lowercase for each sequence name indicates species (h, human; m, mouse; r, rat; dr, *Danio renio* also known as zebrafish). For the sequence name of Gα proteins, a genetic nomenclature was adopted (GNAI, G alpha i; GNAO, G alpha o; GNAQ, G alpha q; GNAS2, G alpha s). The purple shading highlights the conserved sequence compared to the bolded sequence (human $G\alpha_{i3}$). Letters highlighted in blue color are candidate residues for mediating long-range electrostatic interactions. **h**, Schematic representation of the TRPC5-$G\alpha_{i3}$ complex and A_1R-$G\alpha_{i2}\beta\gamma$ complex[4]. The dotted lines and the black line indicate the orientation of α_5H of $G\alpha_i$ in both complexes. Lines were drawn to highlight the relative orientation of two Gα subunits to the membrane (yellowish rectangle) (top). Isopotential contours of molecules are shown (bottom) (−25 mV, red; +25 mV, blue). A reorientation of Gα by tilting approximately 27° after dissociation from a GPCR-G complex is necessary for the positively charged surface of Gα to interact with the negatively charged surface of ARD in TRPC5. **i**, Schematic representation of an isolated $G\alpha_{i3}$ from the TRPC5-$G\alpha_{i3}$ complex is shown. Color reflects the root mean squared deviation (RMSD) between $G\alpha_{i3}$ and isolated $G\alpha_{i2}$ from the A_1R-$G\alpha_{i2}\beta\gamma$ structure[4]. α_5H remained relatively still in both conformations.

Supplementary Table 1. Cryo-EM data collection, refinement and validation statistics

	#1 TRPC5-Gα_{i3} complex, Class1 (TRPC5) (EMD-33022)	#2 TRPC5-Gα_{i3} complex (ARD-Gα_{i3}) (EMD-33022, EMD-34301)	#3 TRPC5-Gα_{i3} complex, Class1 (composite[b]) (EMD-33022) (PDB 7X6I)	#4 TRPC5 in lipid nanodiscs, Class1 (EMD-33021) (PDB 7X6C)	#5 TRPC5-Gα_{i3} complex, Class2 (TRPC5) (EMD-34301)	#6 TRPC5-Gα_{i3} complex, Class2 (composite[c]) (EMD-34301) (PDB 8GVX)	#7 TRPC5 in lipid nanodiscs, Class2 (EMD-34300) (PDB 8GVW)
**Data collection and processing**							
Magnification	92,000	92,000		92,000	92,000		92,000
Voltage (kV)	200	200		200	200		200
Electron exposure (e–/Å^2)	40	40		40	40		40
Defocus range (μm)	−1.0 ~ −2.0	−1.0 ~ −2.0		−1.0 ~ −2.0	−1.0 ~ −2.0		−1.0 ~ −2.0
Pixel size (Å)	1.088	1.088		1.086	1.088		1.086
Symmetry imposed	C4	C1		C4	C4		C4
Initial particle images (no.)	3,261,337	3,261,337		1,550,924	3,261,337		1,550,924
Final particle images (no.)	18,935	205,343[a]		105,020	5,344		33,153
Map resolution (Å)	3.54	4.19		3.15	3.79		3.59
FSC threshold	0.143	0.143		0.143	0.143		0.143
Map resolution range (Å)	2.94 – 39.09	3.44 – 44.92		2.65 – 29.37	3.47 – 61.87		3.06 – 46.48
**Refinement**							
Initial model used (PDB code)			7E4T & 2ODE	7E4T		7X6I, 7E4T	7E4T
Model resolution (Å)			3.93	3.24		3.91	3.54
FSC threshold			0.5	0.5		0.143	0.143
Model composition							
Non-hydrogen atoms			32,908	22,632		32,916	22,496
Protein residues			3,968	2,700		3,964	2,684
Ligands			24	24		24	24
B factors (Å^2)							
Protein			178.60	92.32		114.41	82.16
Ligand			128.18	129.44		124.50	78.94
R.m.s. deviations							
Bond lengths (Å)			0.008	0.004		0.004	0.005
Bond angles (°)			1.209	0.703		1.153	0.878

Supplementary Table 1. *continued*

Validation					
MolProbity score	1.49	1.32		1.61	1.21
Clashscore	6.34	3.20		11.54	3.61
Poor rotamers (%)	0.45	0.66		0.68	0.17
Ramachandran plot					
Favored (%)	97.25	96.70		97.85	97.73
Allowed (%)	2.75	3.30		2.15	2.27
Disallowed (%)	0.00	0.00		0.00	0.00

Footnotes for Supplementary Table 1

[a]Particles after symmetry expansion (C4)

[b]Composite map consists of #2 and #1

[c]Composite map consists of #2 and #5

Supplementary References

1 Pettersen, E. F. *et al.* UCSF Chimera--a visualization system for exploratory research and analysis. *J Comput Chem* **25**, 1605-1612 (2004).

2 Smart, O. S., Neduvelil, J. G., Wang, X., Wallace, B. A. & Sansom, M. S. HOLE: a program for the analysis of the pore dimensions of ion channel structural models. *J Mol Graph* **14**, 354-360, 376 (1996).

3 Tang, Q. *et al.* Structure of the receptor-activated human TRPC6 and TRPC3 ion channels. *Cell Res* **28**, 746-755 (2018).

4 Draper-Joyce, C. J. *et al.* Structure of the adenosine-bound human adenosine A1 receptor-Gi complex. *Nature* **558**, 559-563 (2018).

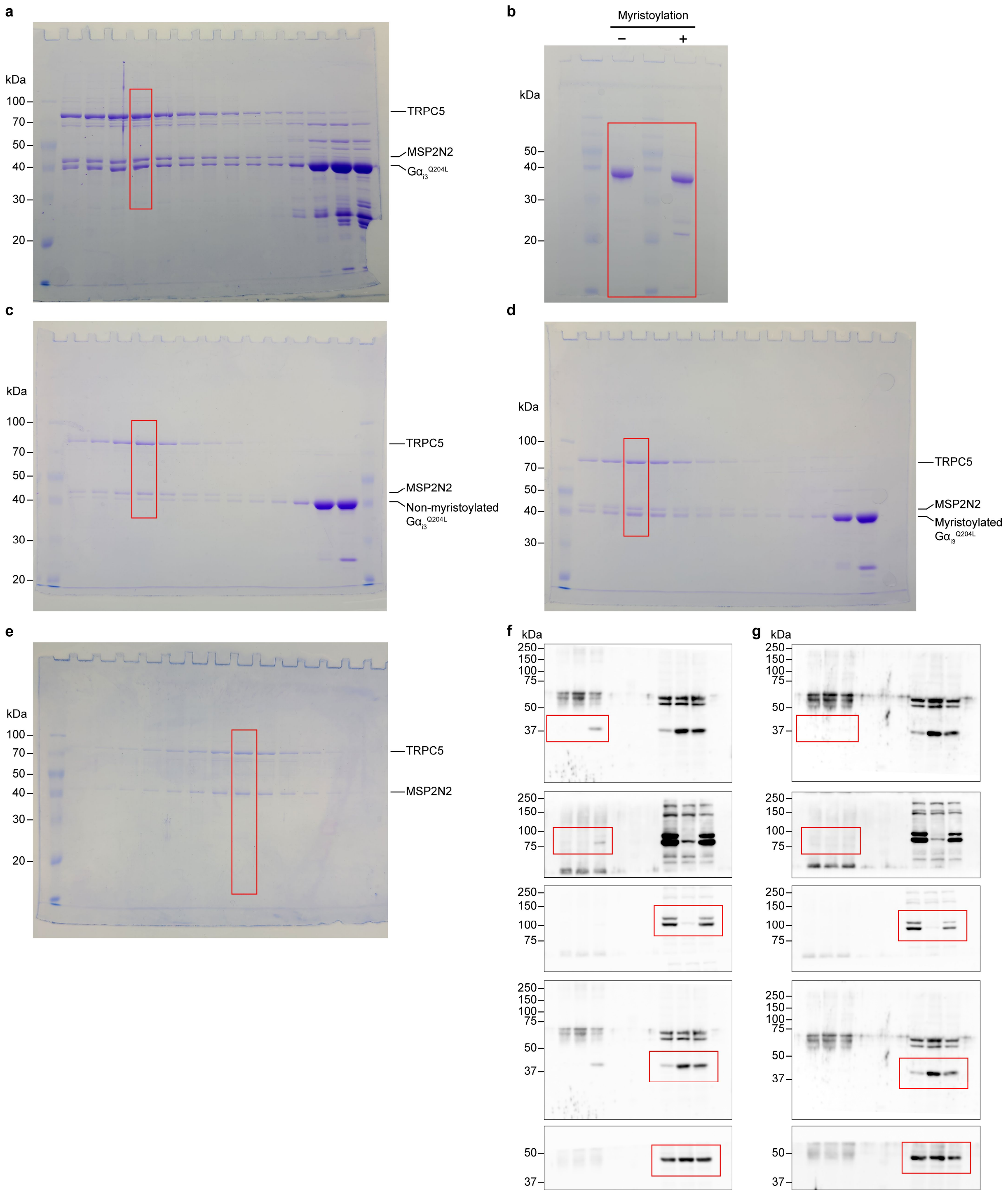

Uncropped gels and blots corresponding to the Supplementary Figs. Cropped regions are indicated as red boxes. **a**, Uncropped gel image for Supplementary Fig. 2d. **b**, Uncropped gel image

for Supplementary Fig. 2e. **c**, Uncropped gel image for Supplementary Fig. 2g, left (non-myristoylated). **d**, Uncropped gel image for Supplementary Fig. 2g, right (myristoylated). **e**, Uncropped gel image for Supplementary Fig. 6b. **f**, Uncropped blot images for Supplementary Fig. 9i, left. **g**, Uncropped blot images for Supplementary Fig. 9i, right.
