# Supplementary material for: Molecular architecture of the Gαi-bound TRPC5 ion channel
Source: Nat Commun. 2023 May 3;14:2550. doi: 10.1038/s41467-023-38281-3 (PMC10156788; doi:10.1038/s41467-023-38281-3)
Supplement: Supplementary file 3 — Description of Additional Supplementary Files [file 41467_2023_38281_MOESM3_ESM.pdf]

File name: Supplementary Movie 1
Description: Conformational dynamics of the TRPC5-Gα_{i3} complex in lipid nanodiscs.

File name: Supplementary Movie 2
Description: Conformational dynamics of the TRPC5 in lipid nanodiscs.

File name: Supplementary Movie 3
Description: Molecular dynamics (MD) simulation of the TRPC5-Gα_{i3} system. A representative video of a 600-ns MD simulation of the TRPC5-Gα_{i3} system, where Gα_{i3} proteins bind stably to the ankyrin repeat domain of the TRPC5. TRPC5 is colored in transparent gray and Gα_{i3} proteins are colored in orange. Only two diagonally opposed subunits of Gα_{i3} are shown for clarity. All water and ion molecules are removed for clarity.

File name: Supplementary Movie 4
Description: Molecular dynamics (MD) simulation of the TRPC5-PIP_2 system. A representative video of a 600-ns MD simulation of the TRPC5-PIP_2 zoomed at one of four binding sites of PIP_2. PIP_2 is colored in black, neighboring positively charged residues are colored in cyan, and other regions in TRPC5 are colored in transparent gray.
