# Supplementary material for: Molecular architecture of the Gαi-bound TRPC5 ion channel
Source: Nat Commun. 2023 May 3;14:2550. doi: 10.1038/s41467-023-38281-3 (PMC10156788; doi:10.1038/s41467-023-38281-3)
Supplement: Supplementary file 8 — Reporting Summary [file 41467_2023_38281_MOESM8_ESM.pdf]

nature portfolio

Corresponding author(s): Hyung Ho Lee

Last updated by author(s): Apr 4, 2023

Reporting Summary

Nature Portfolio wishes to improve the reproducibility of the work that we publish. This form provides structure for consistency and transparency in reporting. For further information on Nature Portfolio policies, see our Editorial Policies and the Editorial Policy Checklist.

Statistics

For all statistical analyses, confirm that the following items are present in the figure legend, table legend, main text, or Methods section.

n/a	Confirmed	
☐	☒	The exact sample size (*n*) for each experimental group/condition, given as a discrete number and unit of measurement
☐	☒	A statement on whether measurements were taken from distinct samples or whether the same sample was measured repeatedly
☐	☒	The statistical test(s) used AND whether they are one- or two-sided<br>*Only common tests should be described solely by name; describe more complex techniques in the Methods section.*
☒	☐	A description of all covariates tested
☒	☐	A description of any assumptions or corrections, such as tests of normality and adjustment for multiple comparisons
☐	☒	A full description of the statistical parameters including central tendency (e.g. means) or other basic estimates (e.g. regression coefficient) AND variation (e.g. standard deviation) or associated estimates of uncertainty (e.g. confidence intervals)
☐	☒	For null hypothesis testing, the test statistic (e.g. *F*, *t*, *r*) with confidence intervals, effect sizes, degrees of freedom and *P* value noted<br>*Give P values as exact values whenever suitable.*
☒	☐	For Bayesian analysis, information on the choice of priors and Markov chain Monte Carlo settings
☒	☐	For hierarchical and complex designs, identification of the appropriate level for tests and full reporting of outcomes
☐	☒	Estimates of effect sizes (e.g. Cohen's *d*, Pearson's *r*), indicating how they were calculated

Our web collection on statistics for biologists contains articles on many of the points above.

Software and code

Policy information about availability of computer code

Data collection	Cryo-EM data collection was performed using EPU 2.10.0.5REL. MD simulation data were collected using OpenMM and Anton2. Commercial software or open-source software was used in all cases; specifically, pClamp 10.7, 11.1 and MetaFlour 7.10.
Data analysis	The following softwares were used in this study: pClamp 10.7, 11.1, MetaFluor 7.10, Relion 4.0, CryoSPARC 3.2.0, Coot 0.9.6, Phenix 1.19, UCSF Chimera 1.15, UCSF ChimeraX 1.3, PyMol, VMD, CHARMM, CHARMM-GUI, Python, Graphpad Prism 9, OriginPro 2016, HOLE 2.0, and AutoDock Vina v1.1.2.

For manuscripts utilizing custom algorithms or software that are central to the research but not yet described in published literature, software must be made available to editors and reviewers. We strongly encourage code deposition in a community repository (e.g. GitHub). See the Nature Portfolio guidelines for submitting code & software for further information.

Data

Policy information about availability of data

All manuscripts must include a data availability statement. This statement should provide the following information, where applicable:

- Accession codes, unique identifiers, or web links for publicly available datasets
- A description of any restrictions on data availability
- For clinical datasets or third party data, please ensure that the statement adheres to our policy

The cryo-EM density maps and corresponding atomic coordinates for the TRPC5Class1, TRPC5Class1-Gαi3, TRPC5Class2 and TRPC5Class2-Gαi3 have been deposited

in the Electron Microscopy Data Bank (EMDB) and Protein Data Bank (PDB) under EMDB accession codes EMD-33021 [https://www.ebi.ac.uk/emdb/EMD-33021], EMD-33022 [https://www.ebi.ac.uk/emdb/EMD-33022], EMD-34300 [https://www.ebi.ac.uk/emdb/EMD-34300], EMD-34301 [https://www.ebi.ac.uk/emdb/EMD-34301] and under PDB accession codes 7X6C [https://doi.org/10.2210/pdb7X6C/pdb], 7X6I [https://doi.org/10.2210/pdb7X6I/pdb], 8GVW [https://doi.org/10.2210/pdb8GVW/pdb], 8GVX [https://doi.org/10.2210/pdb8GVX/pdb], respectively. PDB entries 7E4T [http://doi.org/10.2210/pdb7E4T/pdb] (human TRPC5 apo state structure at 3 angstrom) and 2ODE [http://doi.org/10.2210/pdb2ODE/pdb] (crystal structure of the heterodimeric complex of human RGS8 and activated Gi alpha 3) were used to generate initial templates for model building. The MD simulation data generated in this study have been deposited in the Zenodo OpenAIRE database under accession code 7768238 [https://zenodo.org/record/7768238#.ZB3iqOyZPqY].

Human research participants

Policy information about studies involving human research participants and Sex and Gender in Research.

Reporting on sex and gender	Not applicable.
Population characteristics	Not applicable.
Recruitment	Not applicable.
Ethics oversight	Not applicable.

Note that full information on the approval of the study protocol must also be provided in the manuscript.

Field-specific reporting

Please select the one below that is the best fit for your research. If you are not sure, read the appropriate sections before making your selection.

☒ Life sciences ☐ Behavioural & social sciences ☐ Ecological, evolutionary & environmental sciences

For a reference copy of the document with all sections, see nature.com/documents/nr-reporting-summary-flat.pdf

Life sciences study design

All studies must disclose on these points even when the disclosure is negative.

Sample size	No calculations were made to predetermine the sample size. Sample size was chosen based on literature review, consideration of limited or rare resources, and the number of independent experiments required for strong statistical power and strong inference of hypothesis testing for meaningful conclusions. For cryo-EM, 5,590 and 5,523 movies were obtained using Glacios equipped with a Falcon 4 electron detector for the structure determination of the TRPC5-Gα complex and TRPC5, respectively.
Data exclusions	For cryo-EM, data were excluded using standard classification methods in CryoSPARC and Relion to remove bad particle images and reconstructions. For calculation of fold increase in Po by Gαi3 (Fig. 5l), data points in [diC8-PIP2] < 5 μM and the curves encompassing them were deliberately excluded from the analysis to avoid the numerical singularity. Detailed information on the matter can be found in the figure legend.
Replication	More than ten independent experiments on TRPC5-Gα complex and Gα purification (Supplementary Figure 2 and 3), three independent experiments on myristoylation validation (Supplementary Figure 2), three independent experiments on each BLI assay (Supplementary Figure 9). All electrophysiological experiments were replicated and detailed information on the matter can be found in the figure legends.
Randomization	Randomization is not relevant to this structural study, because the single-particle cryo-EM analysis is based on randomly distributed particle subsets and particles for 3D reconstruction were randomly assigned to calculate gold-standard FSC. All patch-clamp recordings are inherently randomized for no bias was allowed in selecting cells. Same principle was followed in live-cell imaging, FRET measurement, and co-immunoprecipitation.
Blinding	Blinding is not relevant to this study as no subjective allocation was involved.

Reporting for specific materials, systems and methods

We require information from authors about some types of materials, experimental systems and methods used in many studies. Here, indicate whether each material, system or method listed is relevant to your study. If you are not sure if a list item applies to your research, read the appropriate section before selecting a response.

Materials & experimental systems

n/a	Involved in the study	
☐	☒	Antibodies
☐	☒	Eukaryotic cell lines
☒	☐	Palaeontology and archaeology
☒	☐	Animals and other organisms
☒	☐	Clinical data
☒	☐	Dual use research of concern

Methods

n/a	Involved in the study	
☒	☐	ChIP-seq
☒	☐	Flow cytometry
☒	☐	MRI-based neuroimaging

Antibodies

Antibodies used

Mouse monoclonal Anti-FLAG Ab(for detection of FLAG-tagged TRPC5); Sigma Aldrich, F3165; Lot #: SLCG2330
Rabbit polyclonal Anti-Gαi3 Ab; Santa Cruz, sc-262, Lot #: C2311
Mouse monoclonal Anti-β-Tubulin Ab; Sigma Aldrich, T4026, Lot # 128M4790V
Anti-FLAG Ab was used to detect TRPC5-Flag and detailed information on dilution ratio can be found in the Methods section.

Validation

All antibodies were used for co-immunoprecipitation assay in Supplementary Fig. 9 of this article. Validations and relevant citations for each antibody could be found in manufacturers' websites.

Eukaryotic cell lines

Policy information about cell lines and Sex and Gender in Research

Cell line source(s)

HEK293 GnTI-, HEK293T, COS-7, HeLa and Sf9 cells were purchased from ATCC.

Authentication

Cells lines have been authenticated by the vendors. No further authentication was performed for commercially available cell lines.

Mycoplasma contamination

Cell lines were certified as testing negative for mycoplasma by the vendors.

Commonly misidentified lines
(See ICLAC register)

No commonly misidentified cell lines were used in this study.

March 2021
